# New structural insights into Fe_2_P_2_O_7_ – unravelling an unresolved dispute and three reversible phase transitions

**DOI:** 10.1107/S2052252525007547

**Published:** 2025-10-09

**Authors:** Berthold Stöger, Matthias Weil, Robert Glaum, Karla Fejfarová, Vaclav Petříček, Michal Dušek, Eugen Libowitzky, Ekkehard Füglein

**Affiliations:** a X-Ray Centre TU Wien Getreidemarkt 9 A-1060Vienna Austria; b Institute for Chemical Technologies and Analytics, Division of Applied Solid State Chemistry TU Wien Getreidemarkt 9/E164-05-1 1060Vienna Austria; c Department of Inorganic Chemistry University of Bonn Gerhard-Domagk-Strasse 1 D-53121Bonn Germany; d Institute of Physics, ASCR v.v.i., Na Slovance 2, 182 21Praha 8, Czechia; e Department of Mineralogy and Crystallography University of Vienna Josef-Holaubek-Platz 2 A-1090Vienna Austria; f Applications Laboratory, NETZSCH-Gerätebau GmbH, Wittelsbacherstraße 42, D-95100Selb, Germany; ESRF, France

**Keywords:** structural phase transitions, superstructures, incommensurately modulated structures, structure redetermination, pyrophosphates, iron(II) coordination number

## Abstract

The debate in the literature whether the triclinic room-temperature crystal structure of iron(II) pyrophosphate (Fe_2_P_2_O_7_) is centrosymmetric or not has been clearly resolved on the basis of new single-crystal X-ray intensity measurements. This study additionally revealed that Fe_2_P_2_O_7_ undergoes three reversible phase transitions between −140 and 190°C, with the modifications denoted with decreasing temperature as β, α_3_, α_2_ and α_1_. The room-temperature form, α_2_-Fe_2_P_2_O_7_, indeed crystallizes in a centrosymmetric but incommensurately modulated structure, a fact that has not been recognized for more than 40 years. For better comparison with the *C*-centred monoclinic thortveitite-type aristotype (space group type *C*2/*m*), the structure of the hettotype α_2_-Fe_2_P_2_O_7_ is described in the superspace group *C*1(αβγ)0 with *a* = 6.6393 (6), *b* = 8.4748 (6), *c* = 4.4839 (3) Å, α = 90.036 (5), β = 103.962 (7), γ = 92.929 (6)° and a modulation wavevector **q** = 0.4489 (3)a* + 0.2517 (3)b* + 0.3646 (3)c*. The α_2_ modification undergoes two phase transitions towards periodic structures. On heating, a triclinic structure described in *C*1 with very similar lattice parameters is realized above 85°C for the corresponding α_3_ modification. It can be considered as the non-modulated basic structure of the α_2_ modification. At about 185°C, α_3_-Fe_2_P_2_O_7_ transforms to the thortveitite-type β modification, which remains stable up to at least 1000°C. On cooling the α_2_ modification, a triclinic structure of the low-temperature α_1_ modification forms below −140°C, which can be considered as a twofold superstructure of the α_3_ modification with **q** = ½a* + ½b* + ½c*. The result of these phase transitions from the thortveitite-type β-modification via the triclinic α_3_ phase and the incommensurately modulated triclinic α_2_ modification to α_1_-Fe_2_P_2_O_7_ is the complete ordering of the pyrophosphate anion in the low-temperature phase with a P—O—P bridging angle of 151.91 (8)°. This ordering is accompanied by the lowering of the coordination number of one half of the Fe^2+^ ions from 6 to 5.

## Abstract 2

Thortveitite-related Fe_2_P_2_O_7_ shows three reversible phase transitions and has an incommensurately modulated crystal structure at room temperature.

## 1. Introduction

Iron(II) pyrophosphate (Fe_2_P_2_O_7_) is known to show multiple reversible phase transitions. Crystals of one modification were grown under high-temperature conditions (*T* > 1100°C) from partially molten samples. The corresponding room-temperature crystal structure has been reported on the basis of single-crystal X-ray data with triclinic symmetry, either with a non-centrosymmetric model in the space group *P*1 [*a* = 5.517 (2), *b* = 5.255 (2), *c* = 4.488 (1) Å, α = 98.73 (2), β = 98.22 (4), γ = 103.81 (2)°, *V* = 122.6 (2) Å^3^, *Z* = 1 (Stefanidis & Nord, 1982 ▸)], or with a centrosymmetric model, using the non-standard setting *C*1 [*a* = 6.649 (2), *b* = 8.484 (2), *c* = 4.488(l) Å, α = 90.4 (3), β = 103.89 (3), γ = 92.82 (3)°, 245.4 (2) Å^3^, *Z* = 2 (Hoggins *et al.*, 1983 ▸)]. Since polymorphism had not been noticed at that time, polymorphic notation (α, β…) (Tolédano *et al.*, 1998 ▸) was originally not used for this modification. Very recently, it was reported that this room-temperature phase, eventually designated α and likewise modelled in *C*1 on the basis of synchrotron powder data, shows two temperature-induced structural phase transitions on heating (Liang *et al.*, 2024 ▸). One is reported to occur at about 73°C from the triclinic room-temperature α form to an intermediate α′ phase (assumed to be incommensurately modulated), and subsequently at about 183°C to a monoclinic β form, which is isotypic with β-Mg_2_P_2_O_7_ and described in space group *B*2_1_/*c* [non-standard setting of space-group type No. 14 with standard setting *P*2_1_/*c* (Calvo, 1967 ▸; Lukaszewicz, 1967*a* ▸)]. Room-temperature α-Fe_2_P_2_O_7_ is a hettotype of the thortveitite aristotype structure [Sc_2_Si_2_O_7_ (Zachariasen, 1930 ▸; Cruickshank *et al.*, 1962 ▸)], which has monoclinic symmetry (*C*2/*m*, *Z* = 2, *a* ≃ 6.6, *b* ≃ 8.6, *c* ≃ 4.6 Å, β ≃ 103°). The thortveitite structure type or derivatives thereof are adopted by many representatives (Foord *et al.*, 1993 ▸). A second polymorph of Fe_2_P_2_O_7_ was prepared by solid-state reactions at 700°C (Parada *et al.*, 2003 ▸). It crystallizes in the space group *P*2_1_/*c* and was designated the γ polymorph due to its isotypism with the γ form of Co_2_P_2_O_7_ (Kobashi *et al.*, 1997 ▸). Compared with the reported α and β modifications of Fe_2_P_2_O_7_, the γ polymorph has a different structural organization and thus shows no direct group–subgroup relationship (Müller & de la Flor, 2024 ▸) to the thortveitite structure or derivatives thereof.

The renewed interest in Fe_2_P_2_O_7_ originates from its role as a precursor material for the preparation of the lithium iron phosphate cathode materials LiFePO_4_ and Li_2_FeP_2_O_7_ (Hu *et al.*, 2008 ▸; Lee *et al.*, 2012 ▸; Barpanda *et al.*, 2012 ▸; Liu *et al.*, 2015 ▸), as a possible impurity phase during charging/recharging of such cathodes (Ong *et al.*, 2008 ▸), as a by-product during oxidative de­hydrogenation reactions employing the catalyst FePO_4_ (Khan *et al.*, 2010 ▸), or as the calcination product of Fe(II) hy­droxy­phosphono­acetate that – under incorporation of ammonia – shows an enhanced proton conductivity (Salcedo *et al.*, 2020 ▸).

In view of these studies, it is even more surprising that the true nature of the stable triclinic room-temperature form of Fe_2_P_2_O_7_ is still not fully clarified, including debates in the literature about the correct space group. The choice of the non-centrosymmetric model was initially based on two statistical methods with respect to the recorded X-ray diffraction data (Stefanidis & Nord, 1982 ▸), whereas it was argued that the X-ray diffraction data of the other study agreed statistically better with a centrosymmetric distribution (Hoggins *et al.*, 1983 ▸). Some years later, the two models were compared, concluding that the centrosymmetric model is favoured over the non-centrosymmetric one, driven both by statistical and crystal-chemical arguments (Baur & Tillmanns, 1986 ▸). The centrosymmetric model was also supported by an infrared spectroscopic study (Baran *et al.*, 1986 ▸). Nonetheless, a conclusive structure model for the triclinic room-temperature α-Fe_2_P_2_O_7_ modification is missing to this day. Moreover, structural studies regarding the thermal behaviour of this modification with respect to possible phase transitions are restricted to the synchrotron powder study mentioned above. However, due to the limited information one can gain from powder diffraction data, the corresponding results of crystal structure modelling and refinement are frequently associated with certain inaccuracies, especially when it comes to incommensurately modulated structures, as was assumed for the reported α′ phase of Fe_2_P_2_O_7_, for which no further structural details were given (Liang *et al.*, 2024 ▸). Since *M*(II)_2_P_2_O_7_ pyrophosphates with divalent cations of comparable size (*M* = Mg, Cr, Co, Ni, Cu, Zn, except *M* = Mn) show at least one phase transition on heating *or* cooling from the thortveitite aristotype structure to modifications with lower symmetry (Isupov, 2002 ▸; Palatinus *et al.*, 2006 ▸), it appeared likely that phase transitions can also occur for iron(II) pyrophosphate in both directions.

In light of the background and problems described above, we have grown single crystals of Fe_2_P_2_O_7_ and report here on the crystal structure refinement of the triclinic room-temperature phase (herein designated α_2_), shining light on the unresolved problem regarding the structure models reported in the literature. Moreover, we could also determine the crystal structures of the corresponding low-temperature (designated α_1_), intermediate high-temperature (α_3_) and high-temperature (β) forms of Fe_2_P_2_O_7_ from single-crystal X-ray data.

## 2. Experimental

### 2.1. Single-crystal growth

Thortveitite-related Fe_2_P_2_O_7_ can be obtained by synproportionation reactions. Pale-greenish to light-brown single crystals were grown with edge lengths up to 4 mm (Fig. S1 in the supporting information) by chemical vapour transport reactions (Gruehn & Glaum, 2000 ▸; Binnewies *et al.*, 2012 ▸) in a one-pot reaction in sealed and evacuated silica ampoules (volume *ca* 18 cm^3^), starting either from stoichiometric mixtures of FePO_4_ and FeP [equation (1)] as described previously (Glaum *et al.*, 1991 ▸), or, alternatively, from mixtures of FeP_3_O_9_, Fe_2_O_3_ and Fe [equation (2)]. For both reactions, I_2_/P mixtures (≃100 mg I_2_, ≃10 mg P per ampoule) were employed as transport agents in a temperature gradient 850 → 750°C [equation (3)]; the crystals grew on the wall of the silica ampoule placed in the colder sink region and were removed with diluted hydro­fluoric acid (5 wt%).
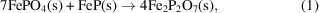

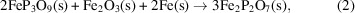

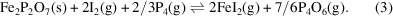
FePO_4_ and FeP_3_O_9_ were prepared in the form of polycrystalline material by heating stoichiometric mixtures of Fe(NO_3_)_3_·9H_2_O (Merck, p.A.) and (NH_4_)_2_HPO_4_ (Fluka, 99%) in a porcelain crucible at 600°C for 24 h; FeP was synthesized in a sealed silica ampoule by reacting iron powder (Fluka, 99%) with red phospho­rus (Knapsack, electronic grade) at 750°C for 36 h with small amounts of iodine (Merck, p.A.) as a mineralizer and a slight surplus of 5% of phospho­rus relative to the stoichiometric amount. All synthesized educts were single phase according to X-ray powder diffraction.

### 2.2. Differential scanning calorimetry

The obtained triclinic α_2_-Fe_2_P_2_O_7_ crystals were finely ground, enclosed in aluminium crucibles with a pierced lid and subjected to a NETZSCH DSC-214 Polyma system in the temperature range −170 to 150°C (26.5 mg sample mass, flowing nitro­gen atmosphere with flow rate 40 ml min^−1^ and a heating/cooling rate of 10°C min^−1^) and to a NETZSCH DSC-200F3 Maia system in the temperature range 30 to 220°C (38.9 mg sample mass, flowing argon atmosphere with flow rate 20 ml min^−1^ and a heating/cooling rate of 20°C min^−1^).

### 2.3. Temperature-dependent powder X-ray diffraction

For temperature-dependent measurements, an HTK1200 Anton-Paar high-temperature furnace chamber was mounted on a PANalytical X’Pert PRO diffractometer (Bragg–Brentano geometry, Cu *K*α radiation, X’Celerator multi-channel detector). The Fe_2_P_2_O_7_ sample was finely ground and placed on a silicon zero-background sample holder. The zero point was previously calibrated with an LaB_6_ standard and was automatically adjusted during the measurements with a PC-controllable alignment stage. The sample was heated under nitro­gen atmosphere at 5°C min^−1^ to the respective temperature and kept for 5 min before measurement of each step to ensure temperature stability; temperature range: 25–1000°C with measurement each 25°C. Refinement of cell parameters was performed with the program *TOPAS* (Bruker, 2009 ▸).

### 2.4. Vibrational spectroscopy

#### 2.4.1. Attenuated total reflectance Fourier-transform infrared spectroscopy

IR powder spectra of α_2_-Fe_2_Si_2_O_7_ were acquired at room temperature from 4000 to 370 cm^−1^ on a Bruker Tensor 27 FTIR spectrometer equipped with a glo(w)bar MIR light source, a KBr beam splitter and a DLaTGS detector. The undiluted sample powder was pressed on the diamond window of a Harrick MVP 2 diamond attenuated total reflectance (ATR) accessory. Sample and background spectra were averaged from 32 scans at 4 cm^−1^ spectral resolution. Background spectra were obtained from the empty ATR unit. Data handling was performed with the *OPUS* 5.5 software (Bruker, 2005 ▸).

#### 2.4.2. Temperature-dependent micro-Raman spectroscopy

Micro-Raman measurements were performed at different temperatures on a confocal micro-Raman spectrometer Renishaw RM1000 equipped with a 20 mW Ar^+^ laser (488 nm) for excitation, an ultra-steep edge filter set facilitating measurements as close as >70 cm^−1^ to the Rayleigh line, a Leica DLML microscope with an Olympus 20×/0.40 ultra-long working distance objective, a 1200 lines mm^−1^ grating in a 300 mm monochromator and a thermo-electrically cooled CCD detector. The entrance slit and CCD readout were set to quasi-confocal mode. The spectral resolution of the system (apparatus function) was 5–6 cm^−1^, and the absolute Raman shift was calibrated by the Rayleigh line and the 521 cm^−1^ line of an Si standard. Spectra were acquired from −30 to 1600 cm^−1^ to employ the Rayleigh line (0 cm^−1^) as an internal standard. Instrument control and data handling was done with *Galactic Grams32* software.

Samples were enclosed in a Linkam FTIR600 heating/cooling stage equipped with thin glass windows and electronic temperature control. A sample crystal was mounted close to the centre of the heated silver block of the stage and covered with a flat silver lid with a centre hole. Thus, the temperature gradient is considered minimal. The acquisition protocol was −150 to 225°C with steps of 25°C. The acquisition time was 120 s at every temperature step.

### 2.5. Single-crystal X-ray diffraction

Intensity data for Fe_2_P_2_O_7_ crystals were collected at 197, 127, 27 and −173°C in a dry stream of nitro­gen on a Stoe STADIVARI diffractometer system equipped with a Dectris Eiger CdTe hybrid photon counting detector using Mo *K*α radiation. The triclinic crystals, *i.e.* those investigated at 127, 27 and −173°C, were generally twinned by monoclinic pseudo-symmetry. By selecting tiny fragments, non-twinned crystals could be isolated and were attached to Kapton micro-mounts. To preclude artefacts in the *F*_obs_ maps due to low spatial resolution, intensity data were collected up to θ ≃ 40°. Data were processed with *X-AREA* (Stoe, 2024 ▸) and integrated with satellites up to the second order for the α_2_ phase. A correction for absorption effects was applied using the multi-scan approach implemented in *LANA* (Koziskova *et al.*, 2016 ▸). An initial model of the α_2_ phase was obtained from charge flipping directly in 3+1-dimensional superspace using *SUPERFLIP* (Palatinus & Chapuis, 2007 ▸). The first refinement cycles of the α_3_ phase were performed using coordinates of the basic structure of the α_2_ phase. The structure of the α_1_ phase was solved using the dual-space approach implemented in *SHELXT* (Sheldrick, 2015 ▸). The structures were refined using *JANA2020* (Petříček *et al.*, 2023 ▸). For the α_3_ and β phases, two refinements were performed for each, one with a linear P_2_O_7_^4−^ group (bridging oxygen atom located on a centre of inversion) and one with the oxygen atom modelled as equally disordered about the centre of inversion.

Experimental details for data collections of the four Fe_2_P_2_O_7_ structures are summarized in Table 1 ▸. Refinement details are compiled in Table 2 ▸ for α_2_-Fe_2_P_2_O_7_ and in Table 3 ▸ for β-, α_3_- and α_1_-Fe_2_P_2_O_7_. Selected bond lengths and angles of the modifications are collated in Table 4 ▸. Further details of the crystal structure investigations may be obtained from the Cambridge Crystallographic Data Centre (CCDC) on quoting the deposition numbers listed at the end of Table 1 ▸. The data can be obtained free of charge via https://www.ccdc.cam.ac.uk/structures.

## 3. Results

### 3.1. Thermal behaviour

**Figure 1:**
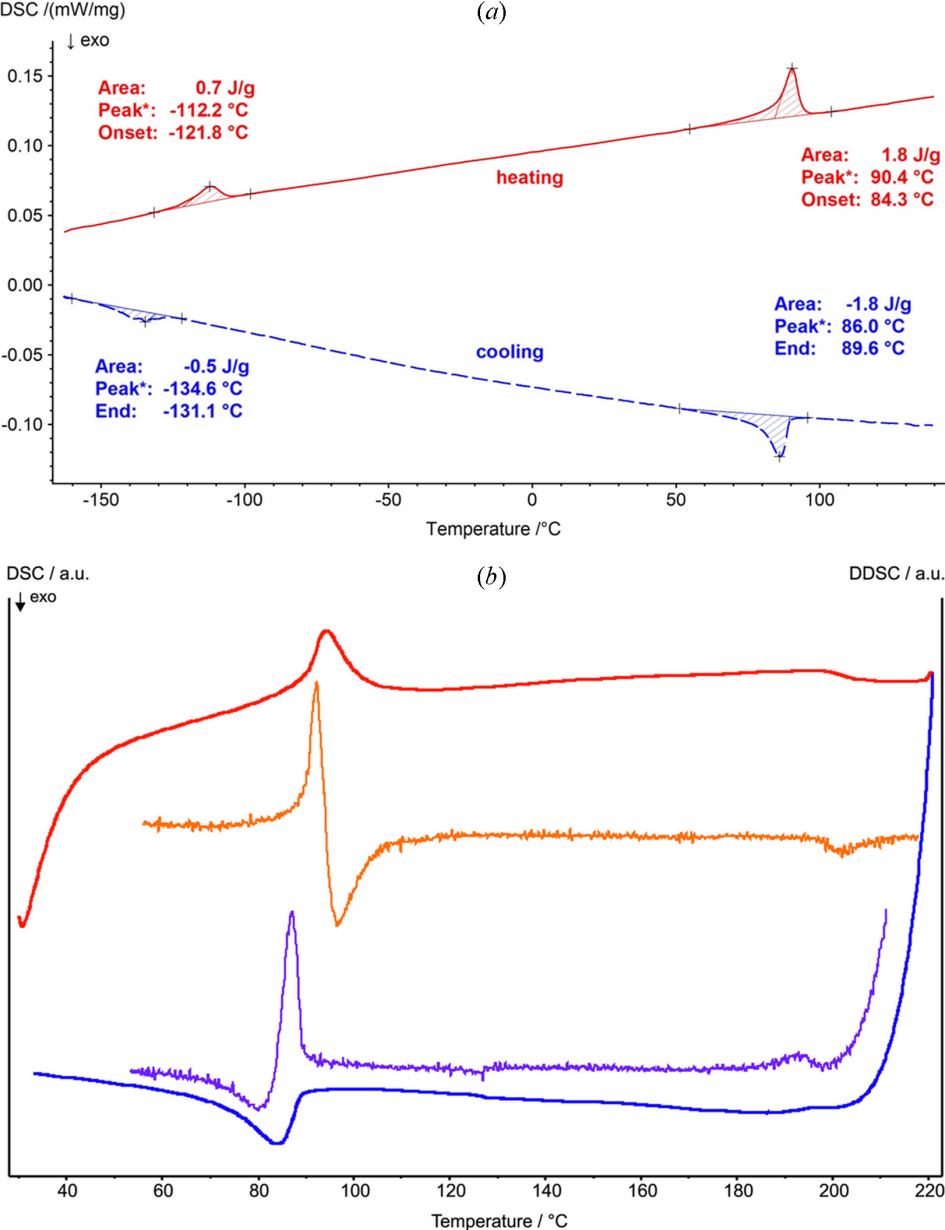
(*a*) DSC curves of Fe_2_P_2_O_7_ in the range −160 to 140°C showing the reversible phase transitions α_1_ ⇌ α_2_ (left) and α_2_ ⇌ α_3_ (right). (*b*) DSC curves of Fe_2_P_2_O_7_ (heating red, cooling blue with curves of the first derivative in orange and lilac, respectively) in the range 30 to 220°C showing the reversible phase transitions α_2_ ⇌ α_3_ (left) and α_3_ ⇌ β (right).

As revealed by a combination of differential scanning calorimetry (DSC), temperature-dependent powder X-ray diffraction (PXRD) and Raman measurements, Fe_2_P_2_O_7_ undergoes three reversible structural phase transitions in the range −120 to 190°C (Figs. 1 ▸–3).

**Figure 2:**
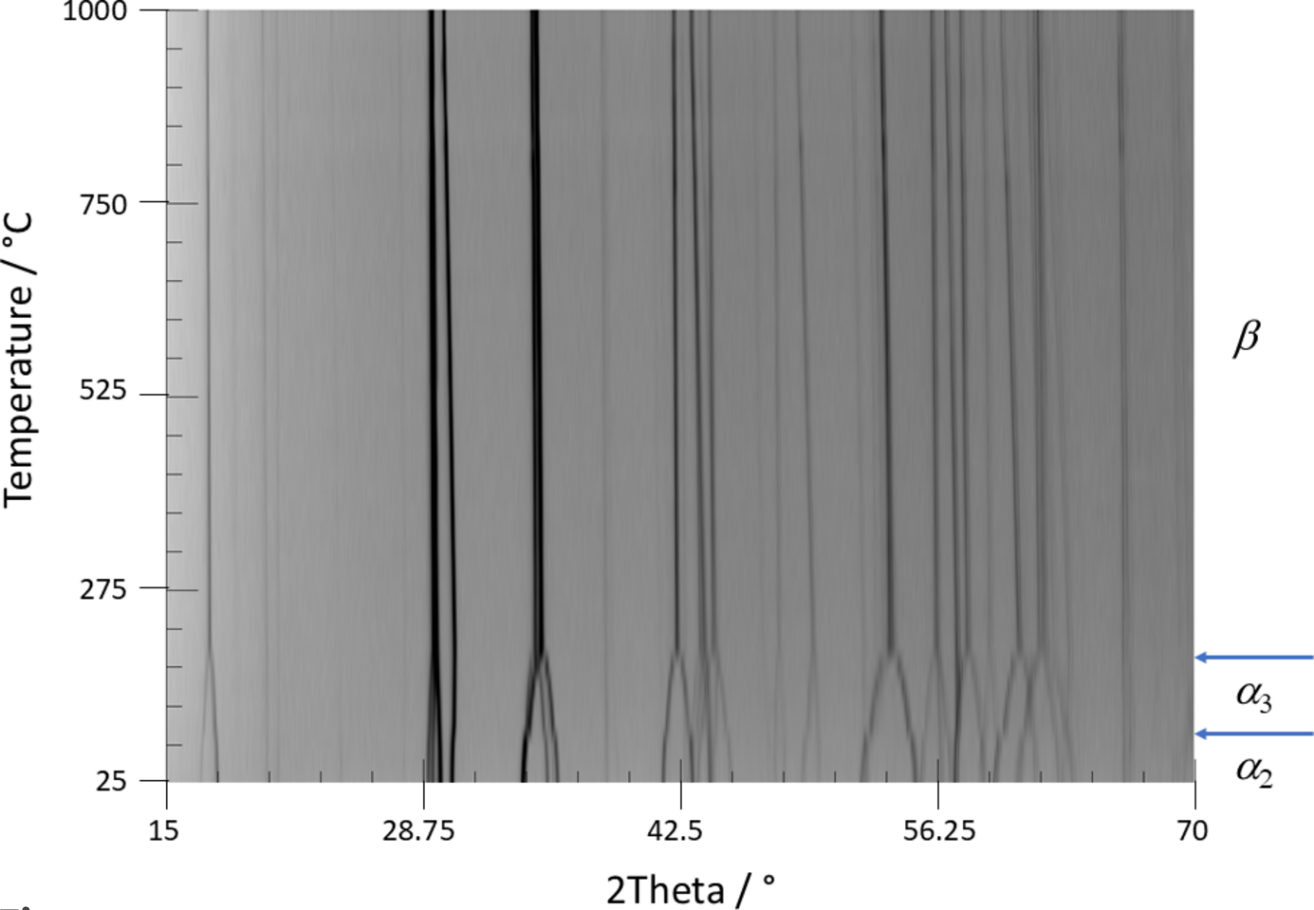
Temperature-dependent PXRD measurements of Fe_2_P_2_O_7_ in the range 25–1000°C with the stability fields of the modifications indicated.

Based on the shapes of the DSC curves, the low-temperature (α_1_) ⇌ room-temperature (α_2_) phase transition is endothermic on heating and exothermic on cooling and proceeds slowly with a somewhat large hysteresis (onset at −121.8°C on heating and at −131.1°C on cooling). The room-temperature (α_2_) ⇌ intermediate high-temperature (α_3_) transition likewise is endothermic on heating and exothermic on cooling but proceeds with a greater transition enthalpy [Fig. 1 ▸(*a*)]. The hysteresis is moderate, with onset temperatures of 84.3°C on heating and at 89.6°C on cooling. The determined temperatures for the latter transition are in a similar range as reported previously (∼73°C; Liang *et al.*, 2024 ▸) and are consistent with the temperature-dependent PXRD data (Fig. 2 ▸), which show a weak but clearly visible step-change in some reflections at around 88°C. The subsequent α_3_ ⇌ β (high-temperature) phase transition is indicated by barely perceptible effects in the DSC curves, seen as very weak shoulders [Fig. 1 ▸(*b*)] with onsets of about 186°C on heating and about 199°C on cooling. This behaviour indicates only very small energetic differences between the α_3_ and β phases. The determined temperatures for the latter phase transition are in rough agreement with the previously reported value of about 183°C (Liang *et al.*, 2024 ▸). On the other hand, the α_3_ ⇌ β phase transition is clearly visible in the temperature-dependent PXRD measurements and is characterized by a convergence of reflections from the triclinic α_3_ phase to the monoclinic high-temperature β form, which is completed at about 190°C (Fig. 2 ▸). The absence of a well resolved peak in the DSC curve and the slow convergence of the lattice parameters indicate a higher order for the α_3_ ⇌ β phase transition, while the other phase transitions are most likely of first order. The evolution of lattice parameters of the α_3_ and β phases with temperature is given in Fig. S2.

**Figure 3:**
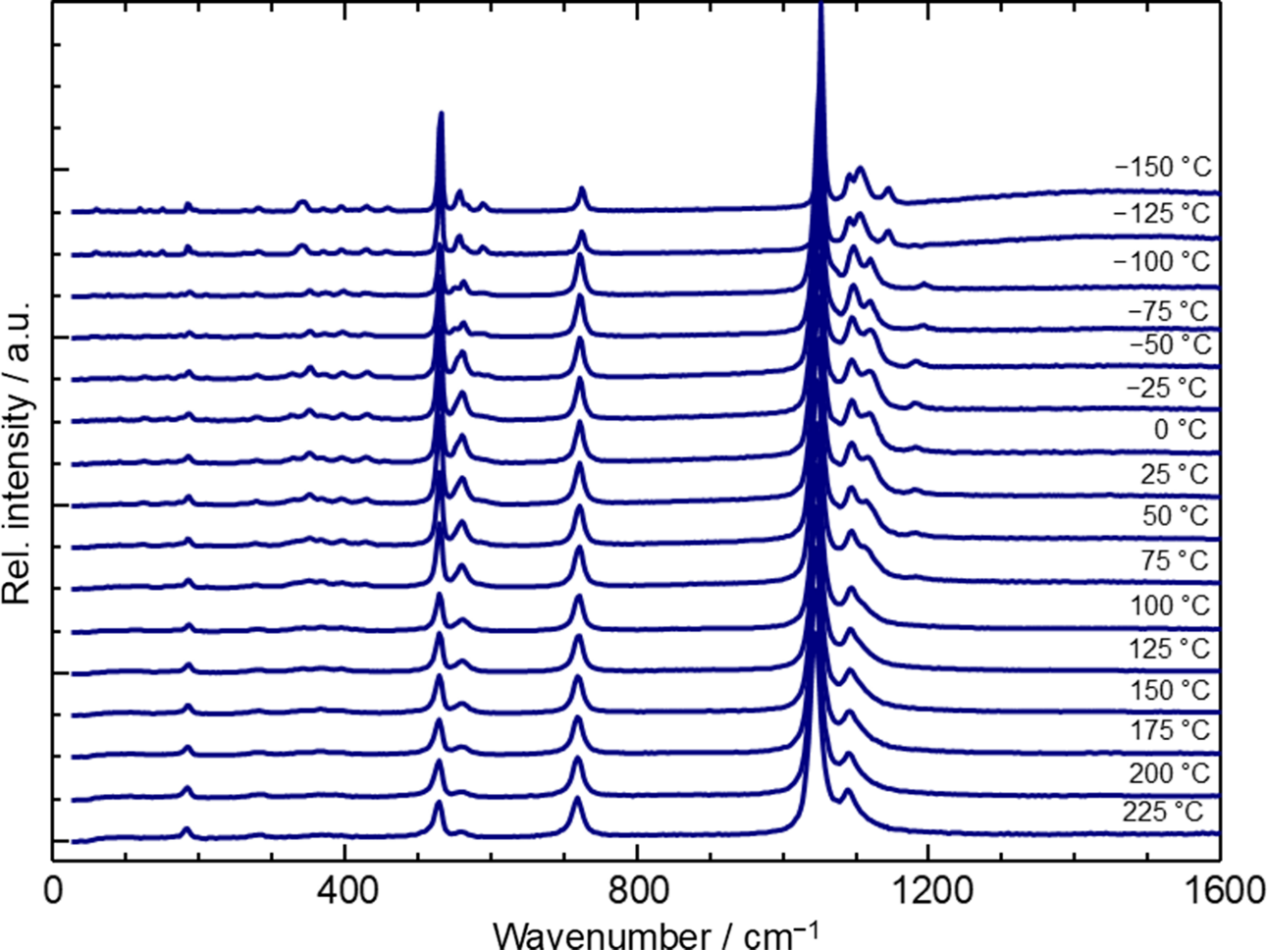
Fe_2_P_2_O_7_. Temperature-dependent Raman spectra for the modifications α_1_ (*T* ≤ −140°C), α_2_ (−140 ≤ *T* ≤ 70°C), α_3_ (70°C ≤ *T* ≤ 190°C) and β (*T* ≥ 190°C).

Raman spectra of Fe_2_P_2_O_7_ have been recorded between −150 and 225°C in 25°C intervals. The observed bands in the spectra vary with temperature (Fig. 3 ▸) and provide additional evidence for the three reversible structural phase transitions already identified by thermal analysis. The continuously increasing number of bands with decreasing temperature also indicates a decrease in the symmetry of the corresponding crystal structures.

**Figure 4:**
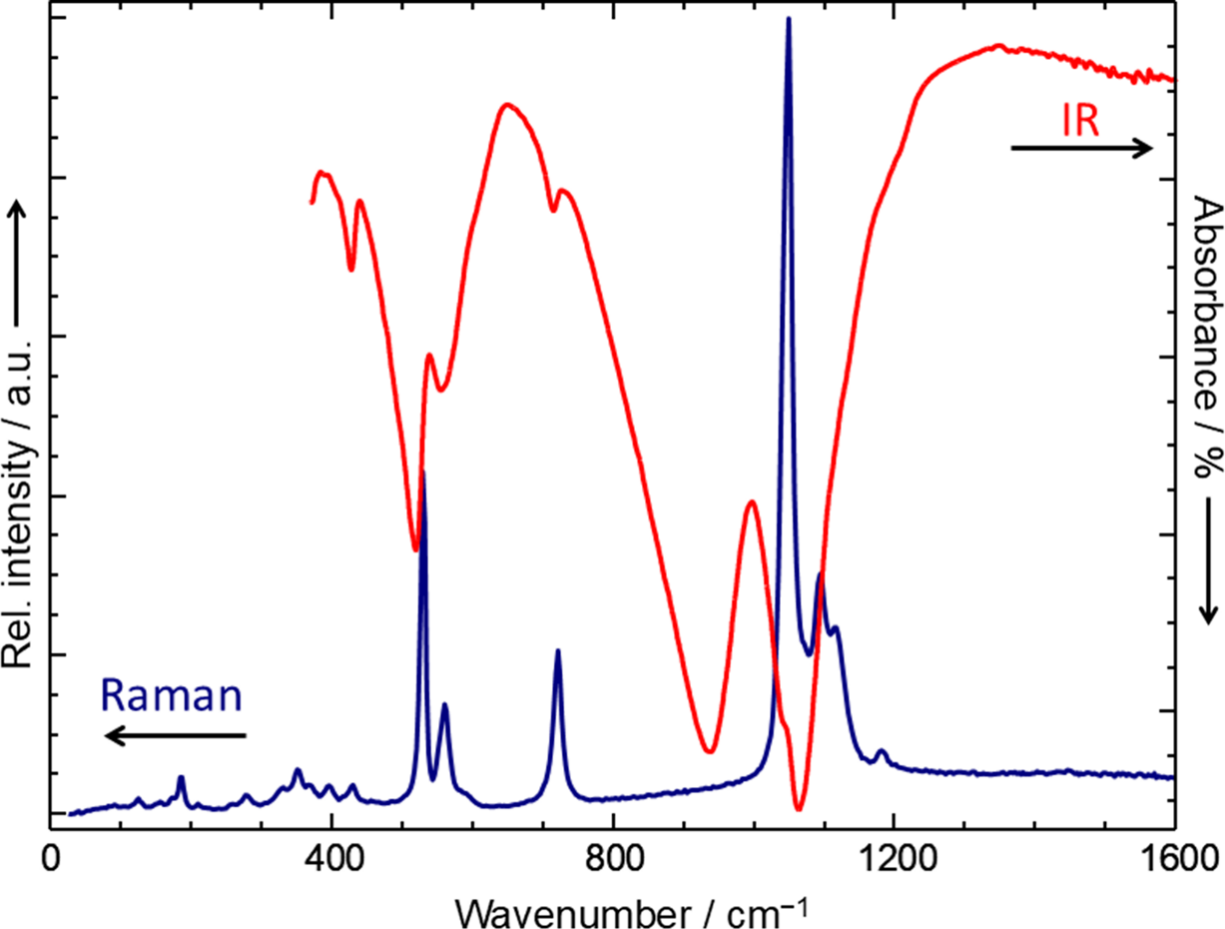
α_2_-Fe_2_P_2_O_7_. IR and Raman spectra at ambient temperature.

The ambient-temperature Raman and IR spectra (Fig. 4 ▸) match those reported in the literature (Baran *et al.*, 1986 ▸). These spectra show the typical signals for metal(II) pyrophosphates (Rulmont *et al.*, 1991 ▸; Popović *et al.*, 2005 ▸). The main spectral features are readily assigned to ν_as_(PO_3_) and ν_s_(PO_3_) (1000 to 1200 cm^−1^), ν_as_(POP) (900 to 1000 cm^−1^), ν_s_(POP) (IR: 714, Raman: 719 cm^−1^), δ_as_(PO_3_) (500 to 600 cm^−1^), and δ_s_(PO_3_) (426 cm^−1^) according to the literature (Baran *et al.*, 1986 ▸). With respect to the focus of this study, the energy, shape and intensity (Raman) of the band related to the symmetric stretching vibration at 719 cm^–1^ deserves particular attention. Apart from a slightly lower relative intensity in the incommensurately modulated α_2_ modification and the commensurately modulated α_3_ modification at −125 and −150°C, respectively (Fig. 3 ▸), this emission shows no variation over the entire temperature range. This suggests that the bond angle and P—O distances in the (P—O—P) core of all pyrophosphate groups in the modifications α_1_, α_2_, α_3_ and β are virtually identical, despite the structural complexity of these modifications described in subsequent sections. In line with observations for iron(II) silicates [orthopyroxene (Mg,Fe)­SiO_3_ (Goldman & Rossman, 1977 ▸); Fe_2_SiO_4_ (Runciman *et al.*, 1973 ▸)], the weak broad hump arising around 1400 cm^–1^ in the Raman spectra of the α_2_ and the α_3_ modifications at the lowest temperatures (Fig. 3 ▸, −125 and −150°C) can be attributed to electronic transitions within the low-symmetry split levels of the ^5^*T*_2*g*_ (ideal *O*_*h*_ symmetry) ground state.

### 3.2. Crystal structures

#### 3.2.1. The thortveitite-type aristotype structure (β-Fe_2_P_2_O_7_) 

**Figure 5:**
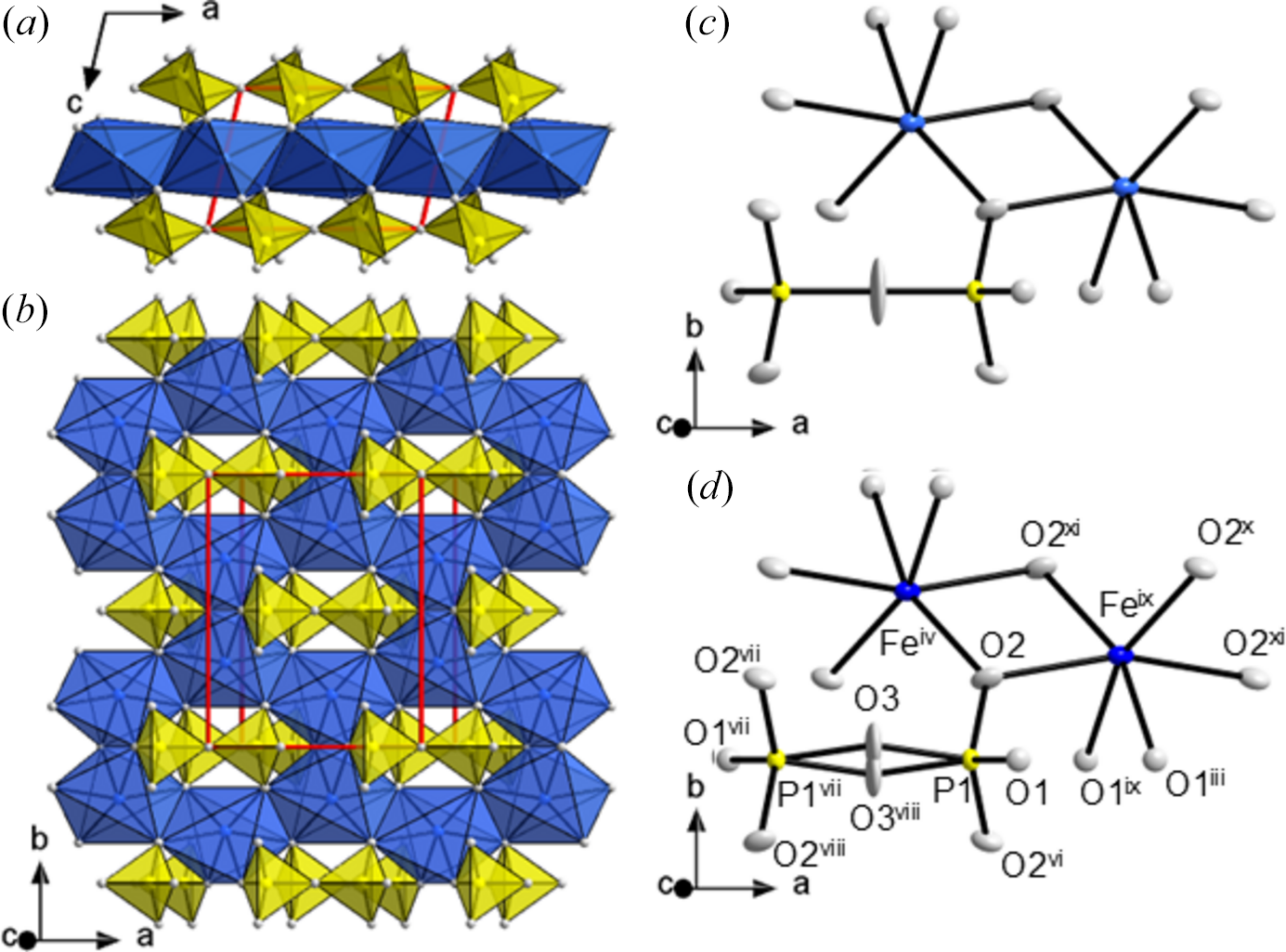
β-Fe_2_P_2_O_7_ (thortveitite-type, *C*2/*m, Z* = 2). Crystal structure with schematic polyhedra [Fe(II)O_6_] (blue) and P_2_O_7_^4−^ groups (yellow) (*a*) viewed along [010] and (*b*) as a projection onto (001); atoms given as spheres of arbitrary radius; the unit cell is indicated in red. *ORTEP* style representations of the polyhedra from refinement of the bridging oxygen atom O3 (*c*) on a single site and (*d*) with split sites; ellipsoids are given at the 50% probability level; symmetry codes refer to Table 4 ▸. [Additional symmetry codes (ix) *x*, *y*, −1 + *z*; (x) *x* + ½, −*y* + ½, *z*; (xi) −*x* + 1, *y*, −*z* − 1; (xii) −*x* + ½, −*y* + ½, −*z* − 1.]

Above 190°C, the β-Fe_2_P_2_O_7_ modification is stable and exists at least up to 1000°C, which was the highest temperature accessible for our measurements. Since monoclinic thortveitite-type β-Fe_2_P_2_O_7_ is the aristotype of the triclinic α_3_-, α_2_- and α_1_-Fe_2_P_2_O_7_ modifications (hettotypes), its crystal structure is discussed first. The basic structural features of β-Fe_2_P_2_O_7_ can be described as made up of alternating layers (metal cations; pyrophosphate anions) parallel to (001) [Fig. 5 ▸(*a*)].

The P_2_O_7_^4−^ anion (composed of two corner-sharing PO_4_ groups) is located on a 2/*m* position [Fig. 5 ▸(*b*)]. Owing to the imposed symmetry, the conformation of the pyrophosphate anion is staggered. The bridging oxygen atom shows enlarged anisotropic displacement parameters (ADPs) and can either be modelled on a single site [Fig. 5 ▸(*c*)] or on split sites with half occupancy [Fig. 5 ▸(*d*)]. Although the crystal structure of the eponymous mineral thortveitite, Sc_2_Si_2_O_7_, is usually described with the bridging oxygen atom located at the 2/*m* position (Cruickshank *et al.*, 1962 ▸), it is still debated whether the bridging oxygen atom in the aristotypic phases should be considered as lying at the 2/*m* position or as dynamically disordered around the mirror plane. Combined neutron/X-ray diffraction studies of thortveitite-type Mn_2_P_2_O_7_ (Stefanidis & Nord, 1984 ▸) corroborate the disorder model. The split-position refinement features slightly better residuals (Table 3 ▸), which is not surprising as the electron density around the origin is modelled with more parameters. A detailed discussion of the differences between a single-site refinement and a split-position refinement is given in the next section for the crystal structure of α_3_-Fe_2_P_2_O_7_. For simplicity, we use the one-site (2/*m*) model to describe the crystal structure of β-Fe_2_P_2_O_7_ in the following. The bond lengths in the P_2_O_7_^4−^ group follow the general trend in pyrophosphate anions (Clark & Morley, 1976 ▸; Durif, 1995 ▸), where the P—O bond lengths to the terminal atoms are shorter [O1: 1.5200 (9) Å, O2: 2 × 1.5205 (8) Å] than to the bridging atom [O3: 1.5596 (4) Å].

Fe^2+^ ions in between the layers of pyrophosphate anions are located on twofold rotation axes. The coordination number of the unique Fe^2+^ ion is 6, and the distorted octahedral coordination polyhedron is defined by the non-bridging atoms of the P_2_O_7_^4−^ anion with four short [2 × 2.0780 (8); 2 × 2.1143 (6) Å] and two longer [2 × 2.3141 (9) Å] Fe—O bonds to the oxygen atoms located at opposite sides. By edge sharing, the [FeO_6_] polyhedra form a honeycomb pattern where one third of the octahedral voids in the resulting layer remain unoccupied [Fig. 5 ▸(*b*)].

#### 3.2.2. The crystal structure of α_3_-Fe_2_P_2_O_7_

On cooling below 190°C, monoclinic β-Fe_2_P_2_O_7_ transforms into a triclinic structure, α_3_-Fe_2_P_2_O_7_, obtained by a *translationengleiche* symmetry descent (Müller & de la Flor, 2024 ▸) of index 2. To simplify the comparison with the known thortveitite phases, the triclinic primitive setting was transformed into the *C*-centred setting of the parent structure, and the relationship of the setting in space group *C*1 to the reduced setting is **a** = −**b**_red_ − **c**_red_, **b** = −**b**_red_ + **c**_red_, **c** = −**a**_red_. The metrical deviation from monoclinic symmetry is most pronounced for the γ angle of 91.616 (7)°.

**Figure 6:**
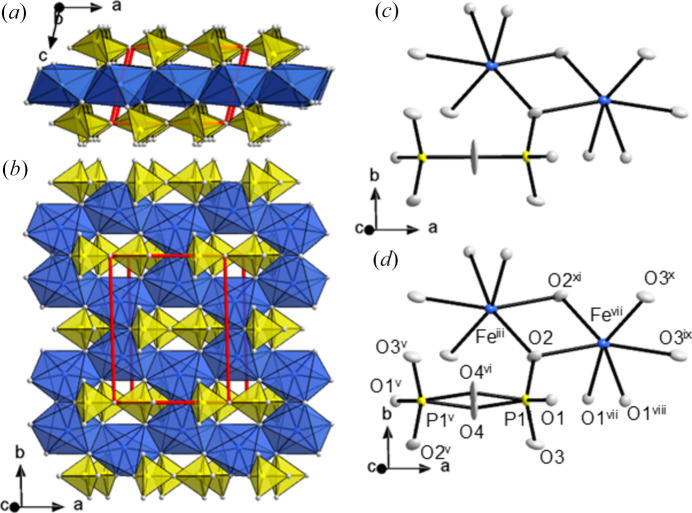
*a*_3_-Fe_2_P_2_O_7_ (*C*1, *Z* = 2). Crystal structure with schematic polyhedra [Fe(II)O_6_] (blue) and P_2_O_7_^4−^ groups (yellow) (*a*) viewed along [010] and (*b*) as a projection onto (001); atoms given as spheres of arbitrary radius; the unit cell is indicated in red. *ORTEP* style representations of the polyhedra from refinement of the bridging oxygen atom O4 (*c*) on a single site and (*d*) with split sites; ellipsoids are given at the 50% probability level; symmetry codes refer to Table 4 ▸. [Additional symmetry codes; (vii) *x*, *y*, *z* − 1; (viii) −*x* + 1, −*y*, −*z*; (ix) −*x* + 1, −*y*, −*z* − 1; (x) *x* + ½, *y* + ½, *z*; (xi) −*x* + ½, −*y* + ½, −*z* − 1]. Note the monoclinic pseudo-symmetry, notably a pseudo-reflection plane parallel to (010) passing through the P_2_O_7_^4−^ unit.

The crystal structure of α_3_-Fe_2_P_2_O_7_ (Fig. 6 ▸) is closely related to that of the aristotype β phase. Owing to the symmetry descent from monoclinic to triclinic, the P_2_O_7_^4−^ unit no longer has 2/*m* symmetry but is located with its bridging atom O4 on an inversion centre; bond lengths and angles in the anion are very similar to the monoclinic modification (Table 4 ▸). The unique Fe^2+^ ion of α_3_-Fe_2_P_2_O_7_ sits on a general position and retains its octahedral coordination polyhedron, however with a larger distortion [2.0377 (10) ≤ *d*(Fe−O) ≤ 2.4021 (13) Å].

**Figure 7:**
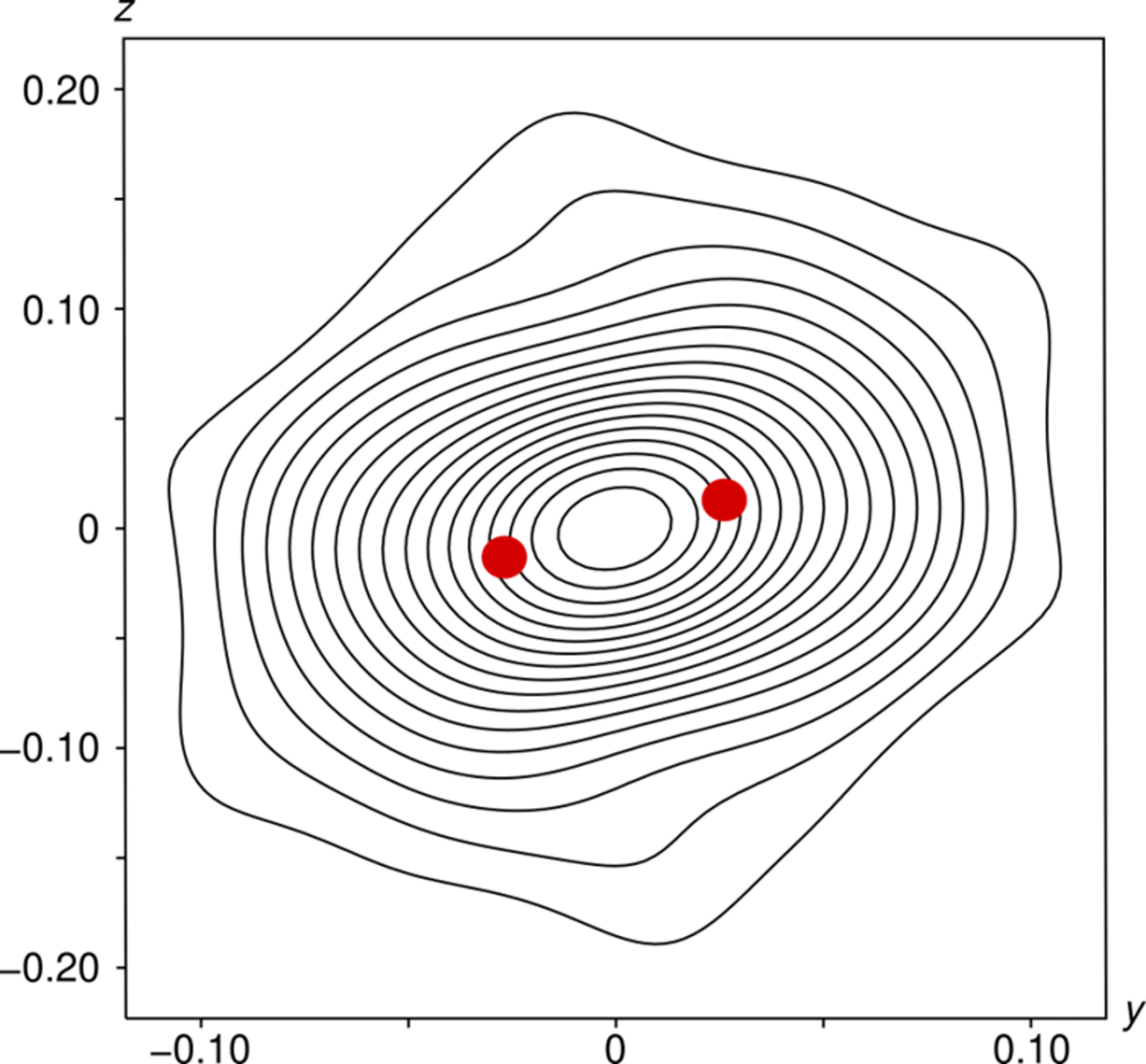
2 × 2 Å^2^ (*y*, *z*) *F*_obs_ contour plot of the electron density resulting from the split-position refinement of α_3_-Fe_2_P_2_O_7_ showing an electron density maximum at the origin, corresponding to a linear P_2_O_7_^4−^ unit. Contours are drawn at the 1 e Å^−3^ levels. Red discs represent the refined position of the O4 atom and its image by inversion at the origin.

Again, the split-position refinement of O4 [Fig. 6 ▸(*d*)] featured slightly better residuals (Table 3 ▸). The highest electron density of the bridging O4 atom is observed at the origin, even in the split-position refinement (Fig. 7 ▸).

For a simplified discussion with regards to the linearity of the pyrophosphate group, we will only consider displacement in the direction of the longest eigenvector of the ADP tensor of bridging atom O4, which is virtually parallel to [001]. As expected, the mean square displacement in that direction is more pronounced for the single-position model with *U* = 0.110 Å^2^, compared with *U* = 0.042 Å^2^ for the split-position model. However, the latter can still be considered as a strong displacement indicative of additional disorder.

**Figure 8:**
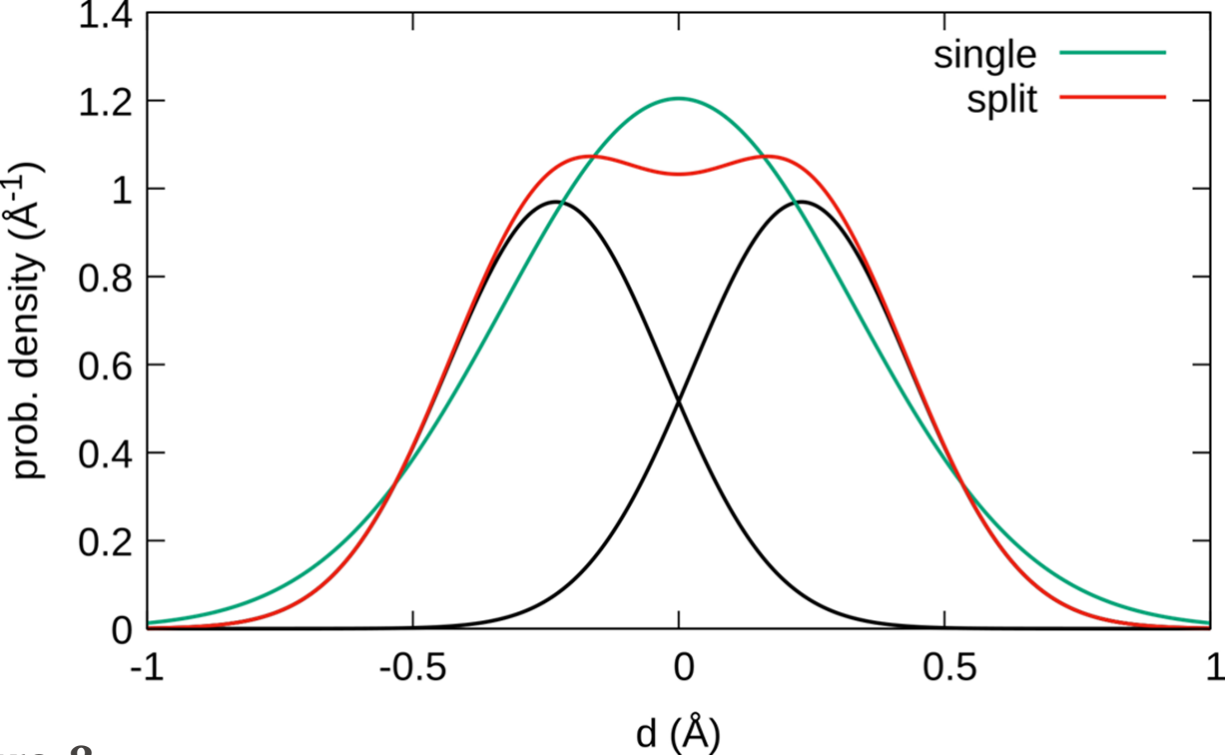
Comparison of one-dimensional normal distributions of the positions of O4 in α_3_-Fe_2_P_2_O_7_ using the largest eigenvalues of the ADP tensor in the single position and in the split-position models. The distributions of the individual atoms in the split-atom model are given in black.

To show the issues in interpreting such models, Fig. 8 ▸ compares the one-dimensional probability distribution of the two models: for the single-position model a normal distribution with the corresponding variance is shown; for the split-position model, two normal distributions with weight ½ spaced by the distance of the two disordered positions are summed. Remarkably, the split-position model features a basically flat distribution at the origin. The small dip at the origin is due to the impossibility of modelling a flat distribution as the sum of two normal distributions and could be remedied by adding an additional position at the origin. It should be stressed that the probability distributions in Fig. 8 ▸ represent the densities of finding the barycentre of the atom at the given position, *not* an electron density distribution. Thus, even though the split-position refinement results in a nominal P—O—P angle of 163.3 (2)°, a linear angle is just as likely according to the refined split-position model. We conclude that in the case of a significant overlap of probability densities, geometric parameters *must not* be derived from atomic positions, as they are meaningless.

In summary, based on the present data, the P_2_O_7_^4−^ group indeed adopts a linear configuration within experimental precision. In fact, configurations with angles *ca* 160–180° appear to possess similar likelihoods. To determine whether linear P_2_O_7_^4−^ units are actually realized, one could resort to high-resolution neutron diffraction experiments, since there the atomic positions are not convolved with diffuse electron clouds.

#### 3.2.3. The crystal structure of α_2_-Fe_2_P_2_O_7_

**Figure 9:**
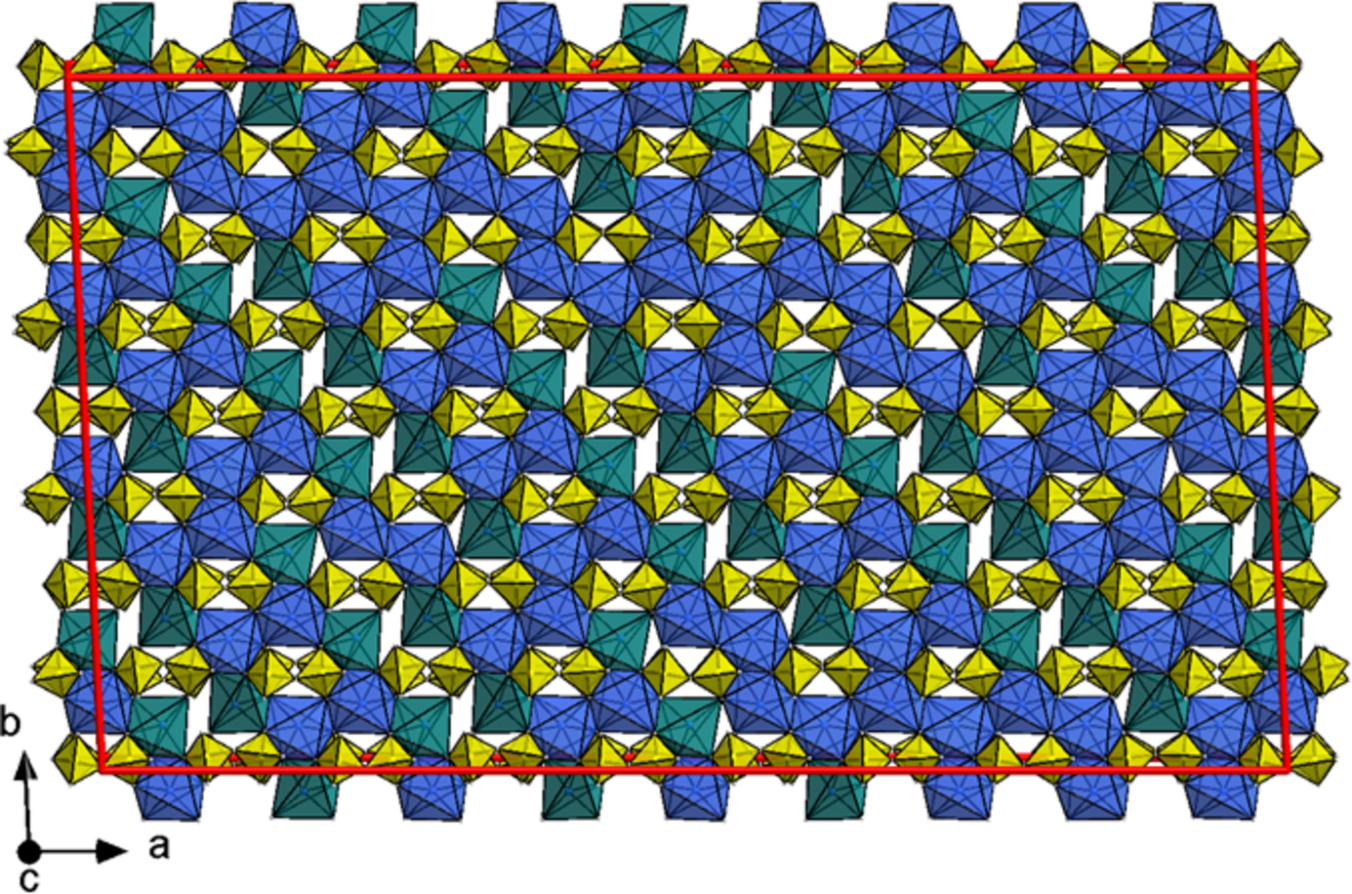
*a*_2_-Fe_2_P_2_O_7_. Section of the crystal structure (–0.03 ≤ *z* ≤ 0.13) in a commensurate approximation (*P*1, *Z* = 792) with schematic polyhedra [Fe(II)O_6_] (blue), [Fe(II)O_5_] (teal) and P_2_O_7_^4−^ groups (yellow) viewed along [001].

The room-temperature phase α_2_-Fe_2_P_2_O_7_ exhibits an incommensurately modulated structure, as shown by the appearance of satellite reflections (Figs. S3 and S4). The basic structure of α_2_-Fe_2_P_2_O_7_, *i.e.* the structure with the reference positions for the modulated structure (Fig. 9 ▸), is the triclinic *C*1 structure of α_3_-Fe_2_P_2_O_7_ (Fig. 6 ▸), and the two structures appear to be virtually identical. Compared with the α_3_ modification, the γ angle [92.929 (6)°] shows an even larger deviation from monoclinic metrics.

The 3+1-dimensional superspace group of α_2_-Fe_2_P_2_O_7_ is *C*1(αβγ)0. In the known monoclinic incommensurately modulated thortveitite-type structures of α_2_-Cr_2_P_2_O_7_ (Palatinus *et al.*, 2006 ▸), α_2_-Zn_2_P_2_O_7_ (Stöger *et al.*, 2014 ▸) or β-Zn_2_As_2_O_7_ (Weil & Stöger, 2010 ▸), the modulation wavevector **q** lies in the (a*, c*) plane for symmetry reasons, which means that translation symmetry is fully retained in the [010] direction. In contrast, α_2_-Fe_2_P_2_O_7_ has a distinct b* component of about one quarter: **q** ≃ 0.449a* + 0.252b* + 0.366c* at 27°C. The vector **q** itself changes with temperature, *e.g.* for a −118°C measurement (*i.e.* shortly before the transition to the α_3_ form) **q** ≃ 0.452a* + 0.291b* + 0.381c*.

To simplify the modelling and discussion of the modulated P_2_O_7_^4−^ group in the α_2_ structure, we placed the origin of the basic structure onto the inversion centre where this group is located. Electron density maps in superspace suggested that the positional modulation of the bridging O4 atom in the incommensurately modulated structure is best described by a function with a single point of discontinuity per period. To both sides of the point of discontinuity, the O4 atom is located at distinct positions without ever adopting any intermediate position (for details, see below).

**Figure 10:**
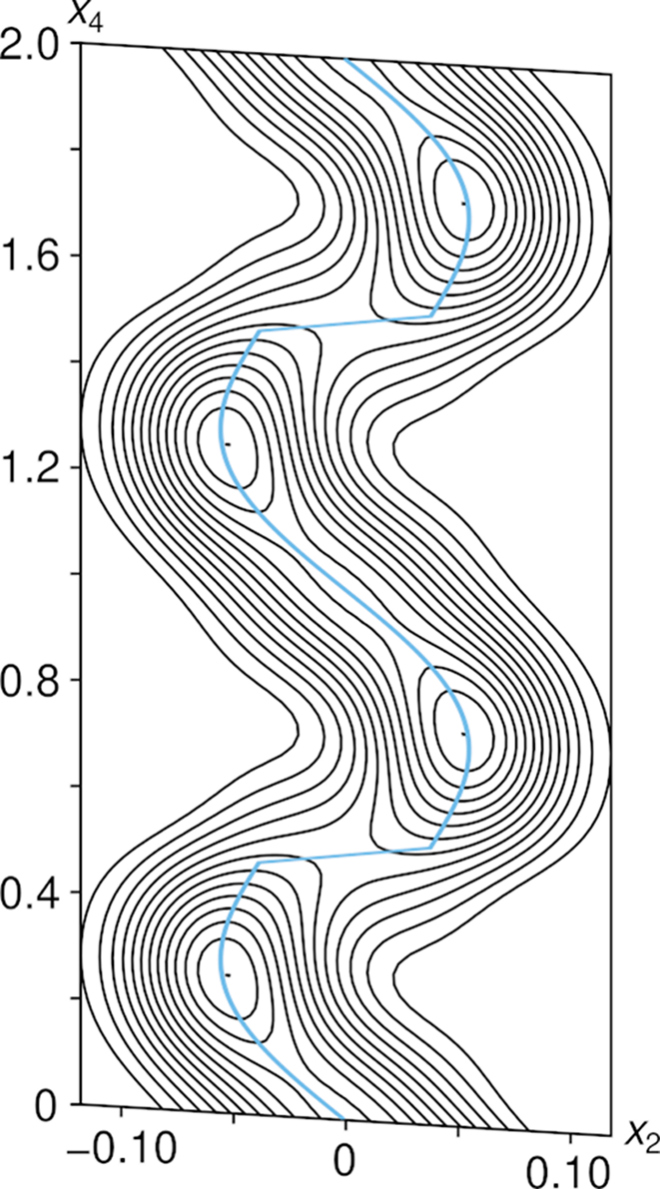
2 Å-wide (*x*_2_, *x*_4_) superspace section at the origin (position of the bridging O4 atom) of α_2_-Fe_2_P_2_O_7_ showing a single point of discontinuity per internal space period at half-integer *t* values. Contours are drawn at the 2 e Å^−3^ levels. The refined centre of gravity of the O4 atom is represented by a blue line. Positional modulation is distinctly less pronounced in the *x*_1_ and *x*_3_ directions and is not shown.

**Figure 11:**
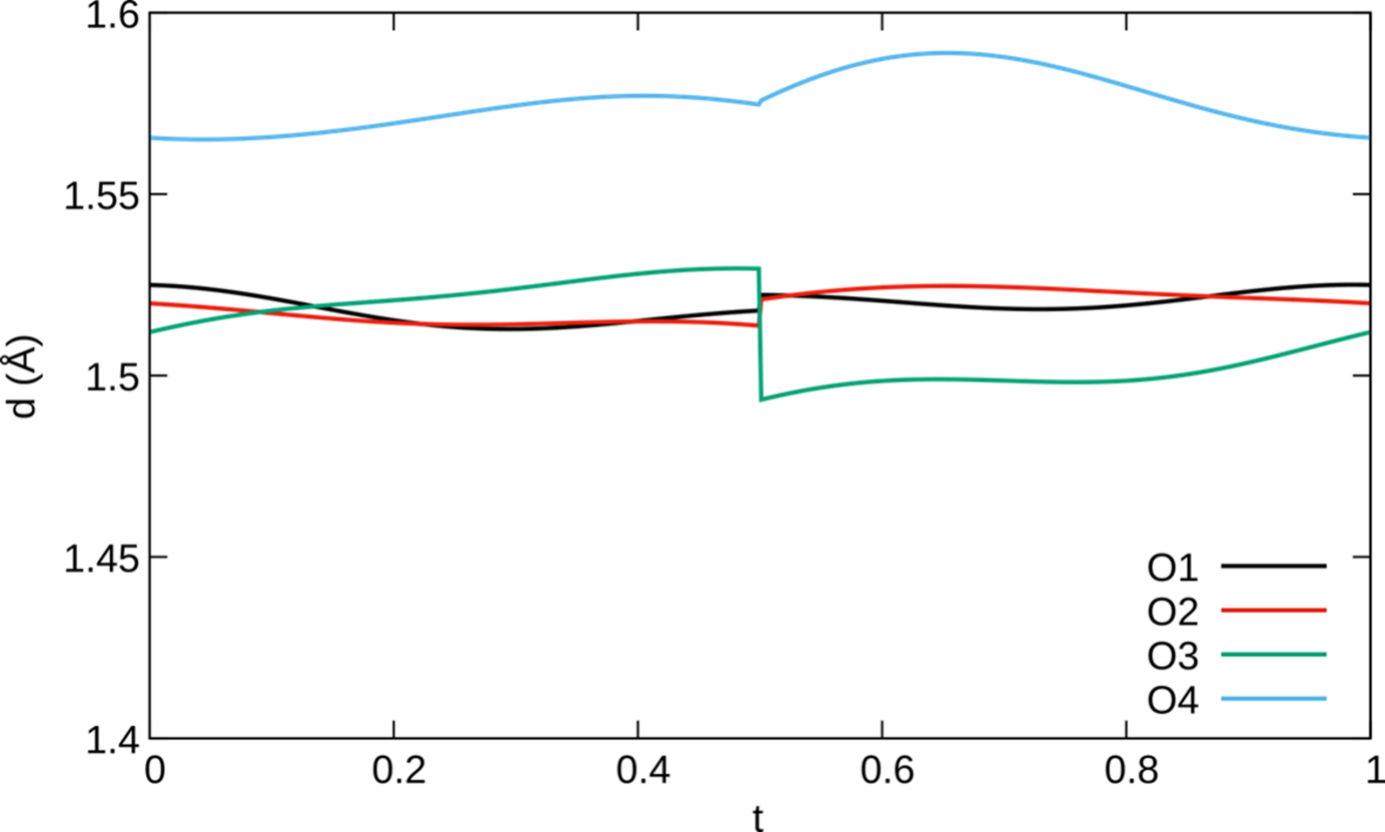
*t*-plot of P—O distances in α_2_-Fe_2_P_2_O_7_ with a point of discontinuity at *t* = ½.

**Figure 12:**
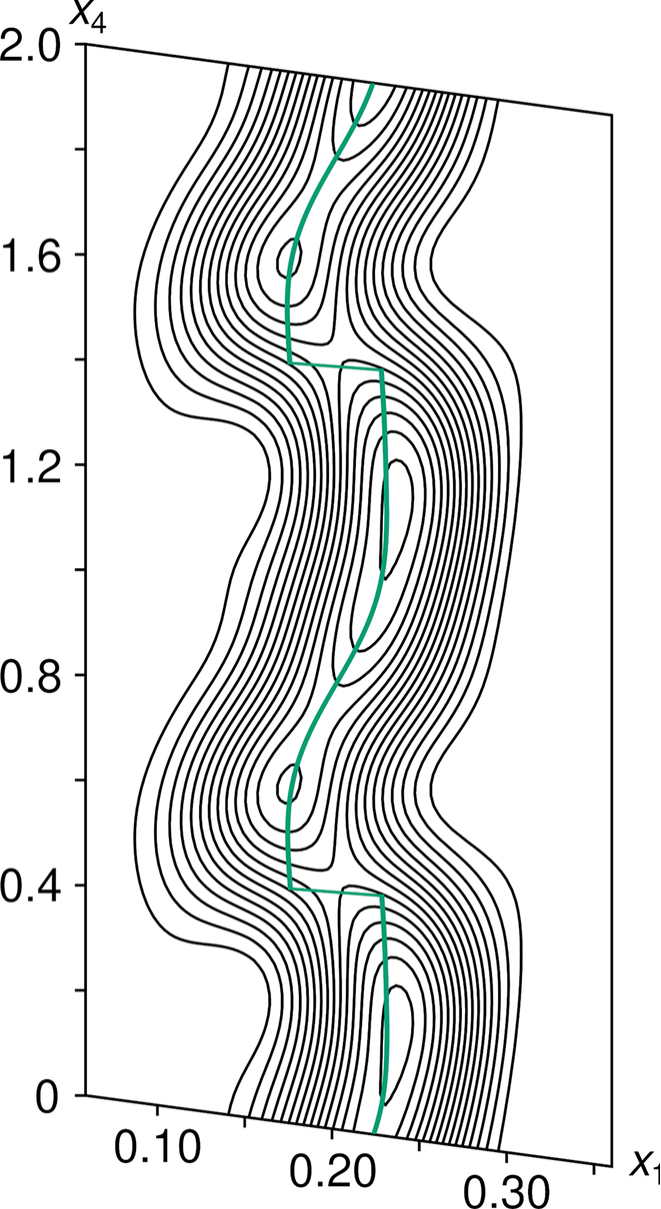
2 Å-wide (*x*_1_, *x*_4_) superspace section of α_2_-Fe_2_P_2_O_7_ centred at the O3 atom showing a single point of discontinuity per internal space period at half-integer *t* values, which is not obvious from the electron density. Contours are drawn at the 2 e Å^−3^ levels. Positional modulation is even more subtle in the *x*_1_ and *x*_3_ directions and is not shown.

The modulation of the bridging oxygen atom (O4) is fundamentally different from the monoclinic incommensurate thortveitites. There, the bridging oxygen atom was modelled using a segment valid for half of the internal space moved away from the 2/*m* position. The other half of the internal space was generated by a symmetry operation acting on 3+1-dimensional superspace. Thus, any *X*_2_O_7_ group in the actual structure is distinctly bent and located to either side of the 2/*m* position, with two points of discontinuity per internal space period. In α_2_-Fe_2_P_2_O_7_, the electron density of O4 in superspace adopts a distinctly different shape and the positional modulation is better described as a sawtooth-like function with a single point of discontinuity per period in the internal space (Fig. 10 ▸). The best results in the sense of giving the most reasonable interatomic distances were obtained by modelling the O4 atom with *x*-harmonics (Petříček *et al.*, 2016 ▸) up to order 2 (4 refined coefficients). To obtain reasonable inter­atomic distances and angles within the PO_4_ groups (Fig. 11 ▸) for all *t* values, the other phosphorus and oxygen atoms needed to be modelled with *x*-harmonics, even though the point of discontinuity was not obvious from superspace electron densities maps (Fig. 12 ▸). The points of discontinuities were fixed at half integer *t*, in accordance with the point of discontinuity of O4. All phosphorus and oxygen atoms were modelled with *x*-harmonics up to second order for positional and displacement parameters.

**Figure 13:**
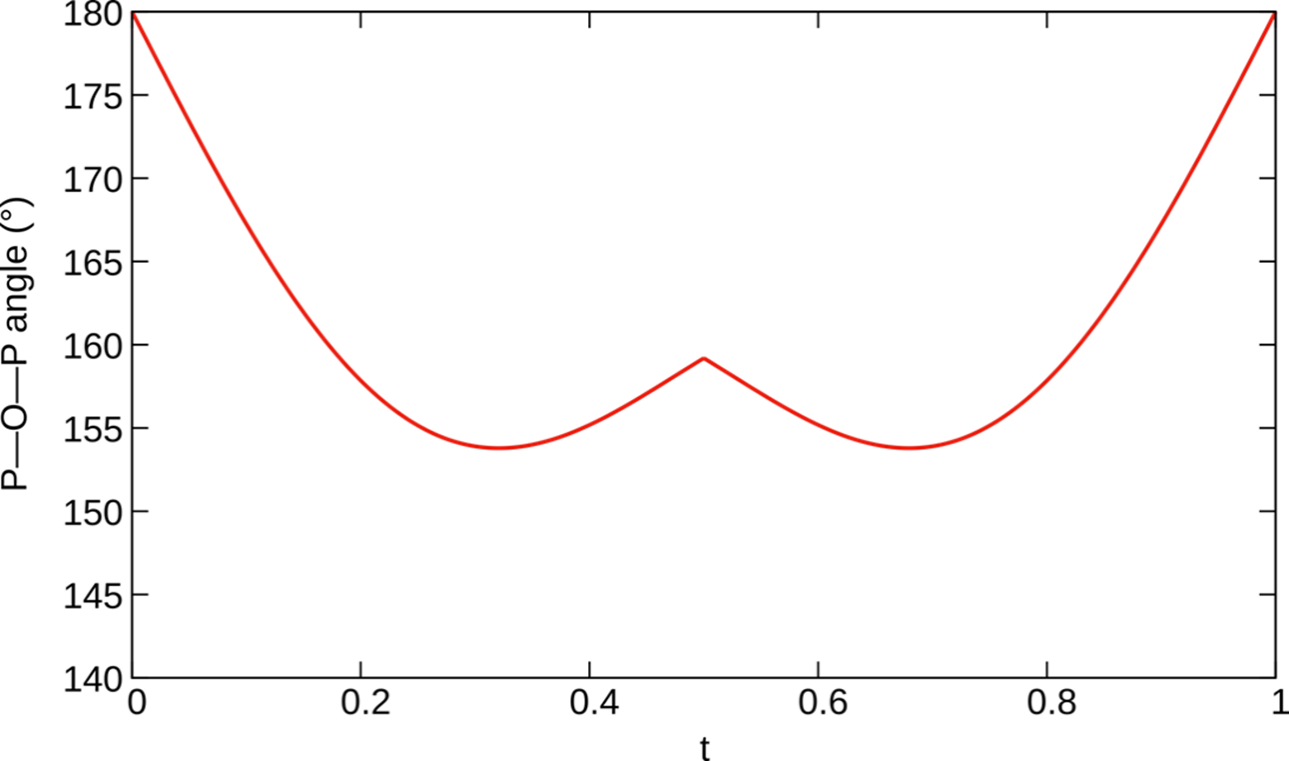
*t*-plot of the P—O—P angle in α_2_-Fe_2_P_2_O_7_, which becomes linear at integral *t*.

**Figure 14:**
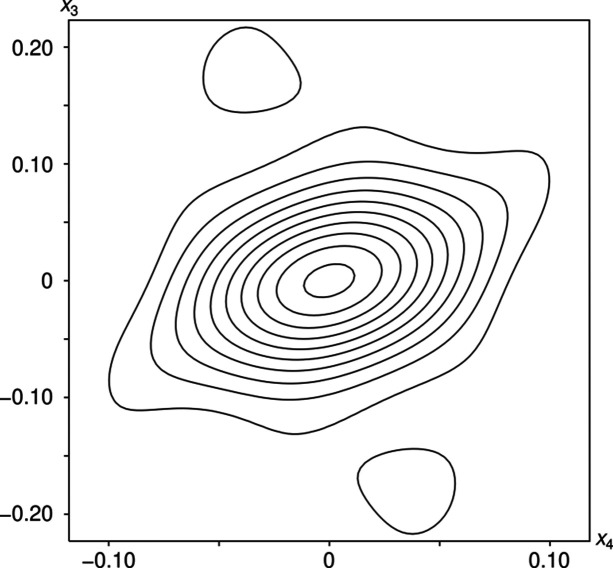
2 × 2 Å^2^ (*x*_2_, *x*_3_) *F*_obs_ contour plot of the electron density of α_2_-Fe_2_P_2_O_7_ at *t* = 0 centred at the origin (position of O4) showing an electron density maximum at the origin, corresponding to a linear P_2_O_7_^4−^ unit. Contours are drawn at the 1 e Å^−3^ levels.

In the given model, the modulation function of the O4 atom passes through the origin at integral *t*, which means that the P—O—P angle becomes arbitrarily close to 180° in parts of the structure (Fig. 13 ▸). In fact, for integral *t*, the highest electron density is observed at the origin (Fig. 14 ▸). Even when introducing a further point of discontinuity at *t* = 0, the two resulting sections of the modulation function converged to nearly meet at the origin.

**Figure 15:**
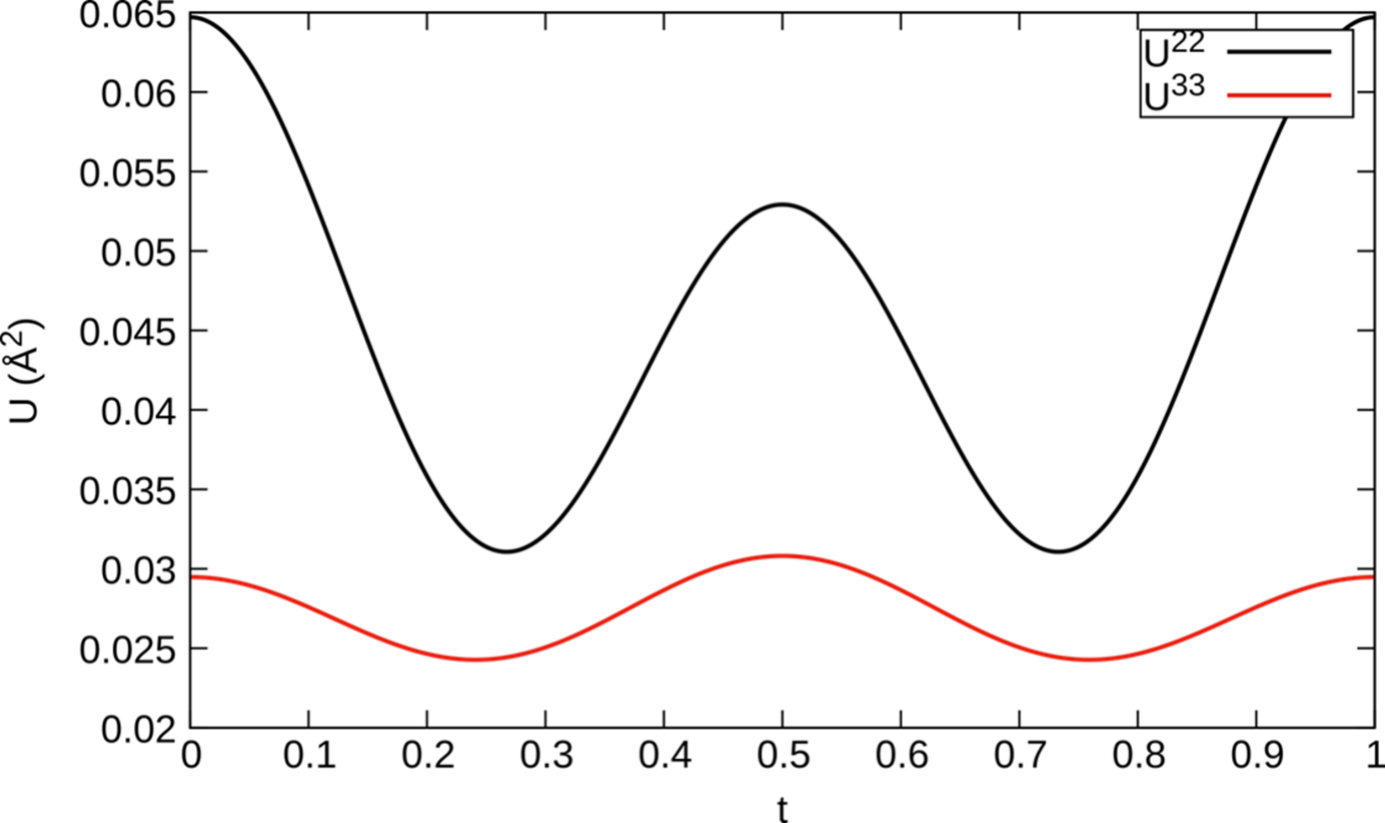
*t*-plot of the *U*^22^ and *U*^33^ ADP tensor elements of the bridging O4 atom in α_2_-Fe_2_P_2_O_7_. The remaining tensor elements are significantly smaller and are not shown.

We assume that a situation similar to the non-modulated α_3_ phase arises at integral *t*, with the *U*^22^ ADP tensor element of O4 being significantly increased at *t* = 0 (Fig. 15 ▸). *U*^22^ describes the displacement of O4 nearly perpendicular to the P—P segment in the [010] direction. Observe that at half-integer *t*, the *U*^22^ element is likewise increased, indicating additional disorder at the point of discontinuity.

**Figure 16:**
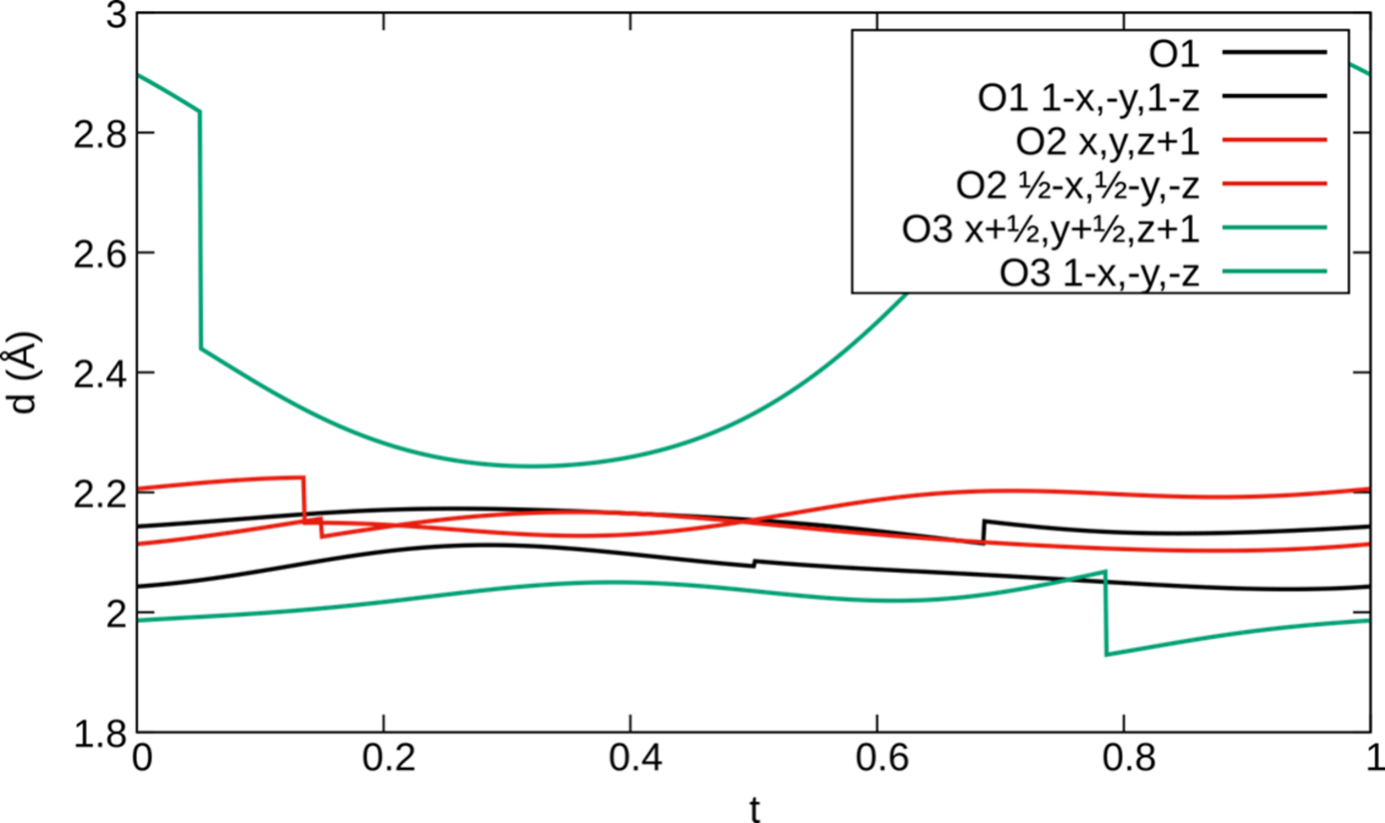
*t*-plot of the Fe−O distances in α_2_-Fe_2_P_2_O_7_ showing distinct regions of fivefold and sixfold coordination.

Another special feature in the known incommensurate thortveitite structures concerns the metal oxide layers and the modulation of the *M*—O distances. In α_2_-Fe_2_P_2_O_7_, the Fe^2+^ ion is five- and sixfold coordinated in different positions of internal space (Fig. 16 ▸), owing to sometimes close and sometimes more distant O3 atoms. The ratio of five- to six-coordination is approximately 1:1. Note that close to the point of discontinuity, the Fe1—O3 bond length of the close O3 atom is unreasonably short. This is most likely a refinement artefact and can be remedied by using *x*-harmonics for Fe1 as well. However, then other Fe1—O distances become too large. In fact, the Fe1 atom connects to six distinct P_2_O_7_^4−^ groups with distinct points of discontinuities in the internal space. Thus, the Fe1 atom would have to be described using six distinct points of discontinuity per internal space period. This appears excessive, and we ultimately applied harmonic modulation functions up to the second order for positional and displacement parameters of the Fe1 atom. Fig. 9 ▸ shows an excerpt of a slab parallel to (001), exemplifying the distribution of fivefold and sixfold coordination around the Fe^2+^ ion. Note that, in contrast to the monoclinic incommensurate thortveitites, translation symmetry is lost in the [010] direction (although it is close to a fourfold superstructure).

#### 3.2.4. The crystal structure of α_1_-Fe_2_P_2_O_7_

The α_1_-Fe_2_P_2_O_7_ modification forms below −140°C and is likewise triclinic. It is a twofold superstructure of α_3_-Fe_2_P_2_O_7_ with the modulation wavevector **q** = ½a* + ½b* + ½c* and obtained by a *klassengleiche* symmetry descent (Müller & de la Flor, 2024 ▸) of index 2. The ‘second-order satellite’ of an *h* + *k* even reflection has again *h* + *k* even and therefore does not violate the reflection condition for the *C*1 space group of α_3_-Fe_2_P_2_O_7_. The modulation wavevector of α_1_-Fe_2_P_2_O_7_ does not relate to that of α_2_-Fe_2_P_2_O_7_, notably with respect to the **b** and **c** components, which are closer to one quarter and one half in α_2_-Fe_2_P_2_O_7_, respectively. The crystal structures of α_1_- and α_2_-Fe_2_P_2_O_7_ are both modulated variants of the α_3_ phase. However, due to the significant change in the modulation vector, it is difficult to classify the α_1_ → α_2_ transition as being of the lock-in type, unlike the other known incommensurate thortveitite-type phases.

To better relate the unit cells of α_1_- and α_3_-Fe_2_P_2_O_7_, the structure of the former is described in the unusual setting (**a**_α__1_, **b**_α__1_, **c**_α__1_) = (2**a**_α__3_, 2**b**_α__3_, ½**a**_α__3_ + ½**b**_α__3_ + **c**_α__3_). In this setting, the additional centring vectors are −¼**a** + ¼**b**, −½**a** + ½**b** and −¾**a** + ¾**b**, and the resulting non-standard centring is represented by the Bravais symbol ‘*X*’. The crystallographic information file (CIF) for α_3_-Fe_2_P_2_O_7_ according to the standard setting in *P*1 is available in the supporting information. The γ angle in the chosen *X*1 setting is directly comparable to that of α_3_ and has moved even further away from 90° than that of the α_2_ modification (Table 1 ▸). The symmetry relationship between α_1_-Fe_2_P_2_O_7_ in *X*1 and α_3_-Fe_2_P_2_O_7_ in *C*1 is of the *isomorphe* type with index 2 (Müller & de la Flor, 2024 ▸). Every second centre of inversion is lost, in particular the point-group symmetry of the P_2_O_7_^4−^ unit is reduced from 1 in the α_3_ modification to 1 in the α_1_ modification. The Fe1 atom of α_3_-Fe_2_P_2_O_7_ is split into two fully occupied positions, Fe1a and Fe1b, in α_1_-Fe_2_P_2_O_7_. In general, split positions are named according to the α_3_-Fe_2_P_2_O_7_ phase with ‘a’ or ‘b’ appended.

**Figure 17:**
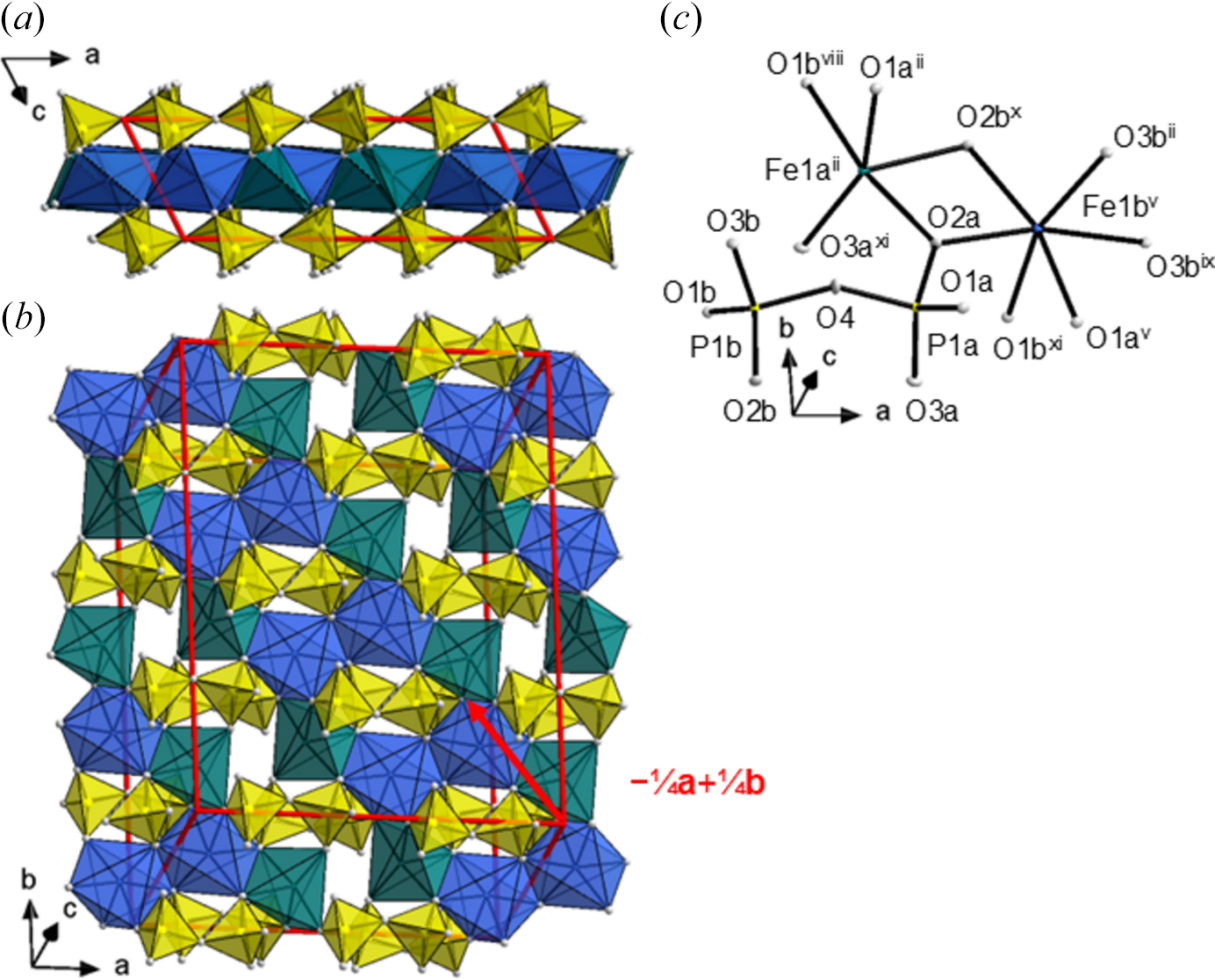
An Fe^2+^ layer and adjacent P_2_O_7_^4−^ layers in α_1_-Fe_2_P_2_O_7_ (*a*) viewed along [010] and (*b*) projected on (001), showing fivefold (teal) and sixfold (blue) coordination of Fe. The unit cell is indicated by red lines. The non-standard −¼**a** + ¼**b** centering translation is indicated by a red arrow. (*c*) *ORTEP* style representations of the polyhedra; ellipsoids are given at the 50% probability level; symmetry codes refer to Table 4 ▸. [Additional symmetry codes: (viii) −*x*, −*y* + 1, −*z* − 1; (ix) *x* + ¾, *y* + ¼, *z* − 1; (x) *x* + ½, *y* + ½, *z* − 1; (xi) −*x* + ¼, −*y* + ¾, −*z* − 1.]

The P_2_O_7_^4−^ group adopts the angle ∠(P—O—P) = 151.91 (8)° and appears in two orientations related by the inversion operation (Fig. 17 ▸). This situation is energetically more favourable than a linear P—O—P angle and is realized in all *M*(II)_2_P_2_O_7_ low-temperature structures: *M* = Mg 144° (Calvo, 1967 ▸); Cr 144.9, 140.1° (Palatinus *et al.*, 2006 ▸); Co 143° (Krishnamachari & Calvo, 1972 ▸); Ni 137° (Lukaszewicz, 1967*b* ▸); Cu 156.8 (Effenberger, 1990 ▸); and Zn 140.6, 148.5° (Stöger *et al.*, 2014 ▸). Again, the averaged values of the P—O_bridging_ bond lengths (average 1.586 Å) are greater than the corresponding values of the P—O_terminal_ bond lengths (average 1.519 Å).

One of the two unique Fe^2+^ ions (Fe1a) has changed its coordination number from six to five, accompanied by an overall shortening of the remaining five Fe—O bond lengths in comparison with six-coordinate Fe^2+^ ions of about 0.05 Å for averaged values. The corresponding coordination polyhedron around Fe1a is a distorted square pyramid [the geometry index τ_5_ = 0.34 (Addison *et al.*, 1984 ▸), where the ideal values for τ_5_ are 0 and 1 for a square pyramid and a trigonal bipyramid, respectively], with the apical atom O2b exhibiting the longest bond of 2.1668 (3) Å in the polyhedron. The ‘next-nearest’ oxygen atom to augment the coordination number of Fe1a to 6 is atom O3a at a distance of 3.1254 (4) Å. This distance is by far too long to be considered as a primary bond because the contribution of this long bond to the bond-valence sum (Brown, 2002 ▸) is only 0.023 valence units using the bond-valence parameters provided by Gagné & Hawthorne (2015 ▸); the calculated bond-valence sum when considering the five close oxygen atoms is 1.932 (2) valence units. The second Fe^2+^ ion (Fe1b) retains octahedral coordination, which appears much more regular than in the high-temperature α_3_ modification; the mean bond length for equatorial oxygen atoms is 2.10 Å, while for axial oxygen atoms it is 2.22 Å. The calculated bond-valence sum for Fe1b is 2.059 (2) Å.

In the α_1_-Fe_2_P_2_O_7_ modification, the sequence of coordination polyhedra along **a** is 5–6–6–5 5–6–6–5 according to the notation introduced by Palatinus *et al.* (2006 ▸), whereby the number represents the coordination number of the central atom, the dash connects two edge-sharing polyhedra and a space separates two neighbouring polyhedra that do not share any atom (Fig. 17 ▸).

## 4. Discussion

To date, Fe_2_P_2_O_7_ is the fourth example of a thortveitite-like structure where incommensurability of one of the modifications occurs, here realized for the room-temperature α_2_ phase intermediate between the commensurate α_3_ and α_1_ phases at higher and lower temperatures, respectively. The other examples are Cr_2_P_2_O_7_ (Palatinus *et al.*, 2006 ▸), Zn_2_P_2_O_7_ (Stöger *et al.*, 2014 ▸) and Zn_2_As_2_O_7_ (Weil & Stöger, 2010 ▸). High-temperature β-Cr_2_P_2_O_7_ crystallizes in the thortveitite aristotype and on cooling subsequently transforms into a structurally unknown modification at 91°C, then into incommensurate α_2_-Cr_2_P_2_O_7_ at 72°C, followed by commensurate α_1_-Cr_2_P_2_O_7_ at 12°C. For Zn_2_P_2_O_7_, a similar temperature-dependent phase transition sequence is observed. High-temperature β-Zn_2_P_2_O_7_ again adopts the thortveitite aristotype structure and transforms in a sluggish phase transition of second order over a wide temperature range into incommensurate α_2_-Zn_2_P_2_O_7_, followed by commensurate α_1_-Zn_2_P_2_O_7_ (isotypic with α_1_-Cr_2_P_2_O_7_) at 135°C. In comparison with the three pyrophosphate phases, Zn_2_As_2_O_7_ is unique as it does not exhibit a high-temperature β-modification with a commensurate structure. Instead, β-Zn_2_As_2_O_7_ has an incommensurately modulated structure and transforms below −6°C into commensurate α-Zn_2_As_2_O_7_ (isotypic with α_1_-Cr_2_P_2_O_7_).

Irrespective of the type of incommensurability or whether the incommensurate phase is intermediate between commensurate high- and low-temperature phases or occurs as a high-temperature form only, a common feature is clearly visible for all four examples. Based on the high-temperature β phases with a coordination number of 6 for the crystallographically unique *M*^2+^ ion, superstructures are realized for the commensurate low-temperature modifications where the crystallographic sites of *M*^2+^ ions are multiplied and now exhibit coordination numbers of 6 or 5. In the (intermediate) incommensurate structures, the coordination of the *M*^2+^ ions varies from 5 to 6, and hence the formation of [*M*O_5_], [*M*O_6_] or [*M*O_5+1_] polyhedra is observed. A change of the coordination number seems not to be the sole reason for the incommensurability in the structures. In fact, the competition between the dynamics of the *X*_2_O_7_ groups due to avoiding of a linear O—*X*—O bridging angle *and* the coordination of the *M*^2+^ ions are decisive for this behaviour. The actual reasons for the change of the coordination number from 6 to 5 still need to be elucidated for the different *M*^2+^ cations. For *M*^2+^ = Cr and Fe with their *d* orbitals not completely filled, electronic effects seem to be the driving forces. Interestingly, Fe_2_P_2_O_7_, which is associated with a weak Jahn–Teller effect of second order for the Fe^2+^ ion (electronic configuration high-spin *d*^6^), has a much lower ordering temperature (≃ −140°C) into the commensurate low-temperature structure than Cr_2_P_2_O_7_, which is associated with a stronger Jahn–Teller effect of first order for Cr^2+^ (electronic configuration *d*^4^). Also, the local coordination environment and distortions of the corresponding [*M*O_5_] and [*M*O_6_] polyhedra in the two commensurate low-temperature structures are different, which leads to a different linkage pattern of 5–6–6–5 5–6–6–5 in α_1_-Fe_2_P_2_O_7_ versus 5–6–5 5–6–5 in α_1_-Cr_2_P_2_O_7_. On the other hand, for *M* = Zn with its completely filled *d* orbitals such electronic effects are missing, and geometric factors or packing features might primarily be responsible for the incommensurability.

The unit-cell volume (Table 1 ▸) of Fe_2_P_2_O_7_ decreases with temperature from α_3_ (123.15 Å^3^ per formula unit at −127°C) to α_2_ (122.25 Å^3^ at 27°C) and α_1_ (121.96 Å^3^ at 127°C) before it increases for β (122.26 Å^3^ at 107°C) and constantly with further temperature (Fig. S2). The associated negative thermal expansion (NTE) observed for the α3, α2 and α1 forms has been studied previously and is attributed to a cooperative Jahn–Teller effect of the Fe^2+^ ions (Liang *et al.*, 2024 ▸). Some other pyrophosphate compounds *A*_2_P_2_O_7_ with *A* cations smaller than 0.97 Å also exhibit NTE in certain temperature ranges (Zeng *et al.*, 2023 ▸), including Cu_2_P_2_O_7_ and Zn_2_P_2_O_7_. In the copper and zinc compounds, however, various phonon modes are described as being responsible for the NTE property (Mochizuki *et al.*, 2024 ▸; Wang *et al.*, 2024 ▸).

## 5. Conclusions

The crystal structures of four different phases of Fe_2_P_2_O_7_ could be unambiguously determined on the basis of new single-crystal X-ray intensity measurements, which also led to a correction of the existence ranges and space-group symmetries of these phases as reported by Liang *et al.* (2024 ▸). It was shown that Fe_2_P_2_O_7_ is already incommensurately modulated under room-temperature conditions [full polymorphic notation (Tolédano *et al.*, 1998 ▸): α_2_|70 to −140°C|*C*1(*αβγ*)0|*Z* = 2|–|Type = hettotype of thortveitite, incommensurately modulated], and both crystal structure models for this triclinic phase reported more than 40 years ago (Stefanidis & Nord, 1982 ▸; Hoggins *et al.*, 1983 ▸) are incorrect. Reversible phase transitions to periodic crystal structures, β|>190°C|*C*2/*m* (15)|*Z* = 2|–|Type = thortveitite, α_3_|190 − 70°C|*C*1 (2)|*Z* = 2|–|Type = hettotype of thortveitite; basic structure of α_2_, and α_1_|<−140°C|*X*1 (2)|*Z* = 8|–|Type = hettotype of thortveitite; twofold superstructure of α_3_, reveal a complex thermal behaviour. Fe_2_P_2_O_7_ is the fourth representative of the thortveitite-type *M*_2_*X*_2_O_7_ family for an incommensurately modulated crystal structure, existing either as an intermediate phase between high- and low-temperature forms (*M* = Cr, *X* = P; *M* = Zn, *X* = P) or as a high-temperature form (*M* = Zn, *X* = As).

We suggest that the incommensurability of the observed α_2_-Fe_2_P_2_O_7_ structure is a result of the competition between the structural distortions caused by the dynamical behaviour of the *X*_2_O_7_ group (avoidance of a linear bridging angle) and the coordination of the metal cations associated with a change in the coordination number from 6 to 5 caused by Jahn–Teller effects.

## Supplementary Material

Crystal structure: contains datablock(s) global, alpha1_standardsetting, alpha1, alpha2, alpha3_single, alpha3_split, beta_single, beta_split. DOI: 10.1107/S2052252525007547/fc5085sup1.cif

Figures S1 to S4. DOI: 10.1107/S2052252525007547/fc5085sup2.pdf

T4fHuXDFsyG

CCDC references: 2482155, 2455104, 2455105, 2455106, 2455107, 2455108, 2455109

## Figures and Tables

**Table 1:** Crystal data and details of data collections

Phase	β-Fe_2_P_2_O_7_	α_3_-Fe_2_P_2_O_7_	α_2_-Fe_2_P_2_O_7_	α_1_-Fe_2_P_2_O_7_
Formula weight	285.60	285.60	285.60	285.60
Diffractometer	Stoe STADIVARI	Stoe STADIVARI	Stoe STADIVARI	Stoe STADIVARI
Radiation, wavelength (Å)	Mo *K*α, 0.71073	Mo *K*α, 0.71073	Mo *K*α, 0.71073	Mo *K*α, 0.71073
Temperature (°C)	197	127	27	−173
Space group (No.)	*C*2/*m* (12)	*C*1 (2)	*C*1(αβγ)0	*X*1 (2)
Centering vectors		(0, 0, 0)*^T^*, (1/2, 1/2, 0)*^T^*	(0, 0, 0, 0)*^T^*, (1/2, 1/2, 0, 0)*^T^*	(0, 0, 0)*^T^*, (−1/4, 1/4, 0)*^T^*, (−1/2, 1/2, 0)*^T^*, (−3/4, 3/4, 0)*^T^*
**q** vector	–	–	0.4489 (3)a* + 0.2517 (3)b* + 0.3646 (3)c*	–
Crystal description	Light-brownish fragment	Light-brownish plate	Light-brownish plate	Light-brownish plate
Crystal dimensions (mm)	0.04 × 0.07 × 0.11	0.01 × 0.04 × 0.06	0.01 × 0.04 × 0.06	0.01 × 0.04 × 0.06
Formula units, *Z*	2	2	2	8
*a* (Å)	6.6154 (6)	6.6161 (5)	6.6393 (6)	13.3690 (5)
*b* (Å)	8.4624 (10)	8.4673 (4)	8.4748 (6)	16.9853 (4)
*c* (Å)	4.4920 (4)	4.4825 (3)	4.4839 (3)	6.3545 (3)
α (°)	90	90.135 (5)	90.036 (5)	50.200 (3)
β (°)	103.502 (7)	103.645 (7)	103.962 (7)	72.409 (4)
γ (°)	90	91.616 (7)	92.929 (6)	94.100 (4)
Volume (Å^3^)	244.52 (4)	243.92 (3)	244.50 (3)	985.17 (11)
μ (mm^−1^)	6.566	6.582	6.566	6.519
Density calc. (g cm^−3^)	3.880	3.889	3.880	3.852
Range θ_min_–θ_max_ (°)	3.98–40.76	3.92–41.04	2.84–37.04	2.80–41.06
Range *h*, *k*, *l*, *m*	−12 → 11,	−11 → 12,	−12 → 11,	−24 → 25,
	−14 → 15,	−9 → 15,	−14 → 13,	−10 → 10,
	−8 → 3	−8 → 8	−6 → 8,	−16 → 16
	–	–	−2 → 2	–
Measured reflections	3414	7257	21588	15018
Independent reflections	826	1563	5785	3198
Observed reflections [*I* > 3σ(*I*)]	815	1341	3402	2830
*R*_i_	0.0119	0.0158	0.0360	0.0161
Absorption correction	Multi-scan	Multi-scan	Multi-scan	Multi-scan
Coef. of transm. *T*_min_, *T*_max_	0.47, 0.56	0.57, 0.69	0.36, 0.55	0.45, 0.54
Structure solution, refinement	–, *JANA2020*	–, *JANA2020*	*SUPERFLIP*, *JANA2020*	*SHELXT*, *JANA2020*
CSD Nos.	2455108 (single site), 2455107 (split site)	2455109 (single site), 2455106 (split site)	2455105	2455104

**Table 2:** Refinement details of the incommensurately modulated α_2_-Fe_2_P_2_O_7_

Phase	α_2_-Fe_2_P_2_O_7_
No. of parameters	251
Extinction coefficient (Becker & Coppens, 1974 ▸)	Isotropic type I, 690 (50)
Difference electron density min, max (e Å^−3^)	−1.15, 1.13
*R*[*F*^2^ > 3σ(*F*^2^)], *wR*(*F*^2^ all)	
all reflections	0.0265, 0.0805
main reflections	0.0241, 0.0834
first-order satellites	0.0264, 0.0641
second-order satellites	0.0606, 0.1767
GooF	1.30

**Table:** 

	β-Fe_2_P_2_O_7_ (split O3)	β-Fe_2_P_2_O_7_ (single O3)
No. of parameters	33	32
Extinction coefficient (Becker & Coppens, 1974 ▸)	Isotropic type I, 320 (90)	Isotropic type I, 330 (100)
Difference electron density min, max (e Å^−3^)	−0.68, 0.58	−0.69, 0.62
*R*[*F*^2^ > 3σ(*F*^2^)], *wR*(*F*^2^ all)	0.0188, 0.0800	0.0194, 0.0825
GooF	1.821	1.878

**Table:** 

	α_3_-Fe_2_P_2_O_7_ (split O4)	α_3_-Fe_2_P_2_O_7_ (single O4)	α_1_-Fe_2_P_2_O_7_
No. of parameters	53	56	101
Extinction coefficient (Becker & Coppens, 1974 ▸)	Isotropic type I, 540 (60)	Isotropic type I, 540 (60)	Isotropic type I, 130 (30)
Difference electron density min, max (e Å^−3^)	−0.18, 0.34	−0.15, 0.37	−1.02, 0.70
*R*[*F*^2^ > 3σ(*F*^2^)], *wR*(*F*^2^ all)	0.0224, 0.0645	0.0218, 0.0627	0.0168, 0.0408
GooF	1.20	1.17	1.28

**Table:** (*a*) β-Fe_2_P_2_O_7_.

Single-site model		Split-site model	
Fe1—O1	2.1143 (6)	Fe1—O1	2.1142 (6)
Fe1—O1^i^	2.1143 (6)	Fe1—O1^i^	2.1142 (6)
Fe1—O2^ii^	2.3141 (9)	Fe1—O2^ii^	2.3141 (9)
Fe1—O2^iii^	2.3141 (9)	Fe1—O2^iii^	2.3141 (9)
Fe1—O2^iv^	2.0780 (8)	Fe1—O2^iv^	2.0782 (8)
Fe1—O2^v^	2.0780 (8)	Fe1—O2^v^	2.0782 (8)
P1—O1	1.5200 (9)	P1—O1	1.5201 (8)
P1—O2	1.5205 (8)	P1—O2	1.5203 (8)
P1—O2^vi^	1.5205 (8)	P1—O2^vi^	1.5203 (8)
P1—O3	1.5596 (4)	P1—O3	1.5759 (7)
P1—O3—P1^vii^	180	P1—O3—P1^vii^	163.5 (3)
		O3⋯O3^viii^	0.453 (6)

**Table:** (*b*) α_3_-Fe_2_P_2_O_7_.

Single-site model		Split-site model	
Fe1—O1	2.0866 (8)	Fe1—O1	2.0868 (8)
Fe1—O1^i^	2.1371 (9)	Fe1—O1^i^	2.1370 (8)
Fe1—O2^ii^	2.2347 (12)	Fe1—O2^ii^	2.2347 (11)
Fe1—O2^iii^	2.1128 (10)	Fe1—O2^iii^	2.1127 (9)
Fe1—O3^iv^	2.0377 (10)	Fe1—O3^iv^	2.0376 (10)
Fe1—O3^v^	2.4021 (13)	Fe1—O3^v^	2.4022 (13)
P1—O1	1.5173 (8)	P1—O1	1.5173 (8)
P1—O2	1.5164 (10)	P1—O2	1.5164 (9)
P1—O3	1.5218 (10)	P1—O3	1.5219 (10)
P1—O4	1.5593 (4)	P1—O4	1.552 (8)
		P1—O4^vi^	1.600 (8)
P1—O4—P1^v^	180	P1—O4—P1^v^	163.3 (3)
		O4⋯O4^vi^	0.461 (7)

**Table:** (*c*) α_2_-Fe_2_P_2_O_7_.

	Average	Minimum	Maximum
Fe1—O1	2.0724 (19)	2.039 (2)	2.112 (2)
Fe1—O1^i^	2.151 (2)	2.119 (2)	2.174 (2)
Fe1—O2^ii^	2.178 (2)	2.130 (3)	2.226 (3)
Fe1—O2^iii^	2.134 (2)	2.104 (2)	2.168 (2)
Fe1—O3^iv^	2.013 (2)	1.930 (2)	2.066 (2)
Fe1—O3^v^	2.550 (3)	2.245 (3)	2.962 (3)
P1—O1	1.5201 (19)	1.504 (2)	1.526 (2)
P1—O2	1.520 (2)	1.481 (2)	1.525 (2)
P1—O3	1.512 (2)	1.493 (2)	1.529 (2)
P1—O4	1.5760 (19)	1.494 (3)	1.592 (3)

**Table:** (*d*) α_1_-Fe_2_P_2_O_7_.

Fe1a—O1a	2.0580 (11)	Fe1b—O1a	2.1742 (8)
Fe1a—O1b^i^	2.1397 (8)	Fe1b—O1b^i^	2.0860 (12)
Fe1a—O2a^ii^	2.1005 (8)	Fe1b—O2a^v^	2.1201 (7)
Fe1a—O2b^iii^	2.1668 (8)	Fe1b—O2b^vi^	2.1903 (8)
Fe1a—O3a^iv^	1.9756 (12)	Fe1b—O3b^vii^	2.0325 (11)
		Fe1b—O3b^iii^	2.2463 (7)
P1a—O1a	1.5254 (9)		
P1a—O2a	1.5238 (8)		
P1a—O3a	1.5033 (12)		
P1a—O4	1.5921 (8)		
P1b—O1b	1.5194 (9)		
P1b—O2b	1.5209 (9)		
P1b—O3b	1.5228 (11)		
P1b—O4	1.5795 (8)		
P1a—O4—P1b	151.91 (8)		

Symmetry codes: (i) *x* + 1/4, *y* − 1/4, *z* + 1; (ii) −*x* + 1/4, −*y* + 3/4, −*z*; (iii) −*x* − 1/4, −*y* + 1/4, −*z* + 1; (iv) *x*, *y*, *z* + 1; (v) −*x* + 1/2, −*y* + 1/2, −*z*; (vi) −*x*, −*y*, −*z* + 1; (vii) *x* + 1/4, *y* − 1/4, *z*.

## References

[bb1] Addison, A. W., Rao, N. T., Reedijk, J., van Rijn, J. & Verschoor, G. C. (1984). *J. Chem. Soc. Dalton Trans.* pp. 1349–1356.

[bb2] Baran, E. J., Botto, I. L. & Nord, A. G. (1986). *J. Mol. Struct.* **143**, 151–154.

[bb3] Barpanda, P., Nishimura, S.-I. & Yamada, A. (2012). *Adv. Energy Mater.* **2**, 841–859.

[bb4] Baur, W. H. & Tillmanns, E. (1986). *Acta Cryst.* B**42**, 95–111.

[bb5] Becker, P. J. & Coppens, P. (1974). *Acta Cryst.* A**30**, 129–147.

[bb6] Binnewies, M., Glaum, R., Schmidt, M. & Schmidt, P. (2012). *Chemical vapor transport reactions*. Berlin: De Gruyter.

[bb7] Brown, I. D. (2002). *The chemical bond in inorganic chemistry: the bond valence model*. Oxford University Press.

[bb9] Bruker (2005). *OPUS*. Version 5.5. Bruker Optik GmbH, Ettlingen, Germany.

[bb8] Bruker (2009). *TOPAS*. Version 4.2. Bruker AXS GmbH, Karlsruhe, Germany.

[bb10] Calvo, C. (1967). *Acta Cryst.* **23**, 289–295.

[bb11] Clark, G. M. & Morley, R. (1976). *Chem. Soc. Rev.* **5**, 269–295.

[bb12] Cruickshank, D. W. J., Lynton, H. & Barclay, G. A. (1962). *Acta Cryst.* **15**, 491–498.

[bb13] Durif, A. (1995). *Crystal chemistry of condensed phosphates*. New York: Plenum Press.

[bb14] Effenberger, H. (1990). *Acta Cryst.* C**46**, 691–692.

[bb15] Foord, E. E., Birmingham, S. D., Demartin, F., Pilati, T., Gramaccioli, C. M. & Lichte, F. E. (1993). *Can. Mineral.* **31**, 337–346.

[bb16] Gagné, O. C. & Hawthorne, F. C. (2015). *Acta Cryst.* B**71**, 562–578. 10.1107/S2052520615016297 PMC4591556 26428406

[bb17] Glaum, R., Walter–Peter, M., Özalp, D. & Gruehn, R. (1991). *Z. Anorg. Allge Chem.* **601**, 145–162.

[bb18] Goldman, D. S. & Rossman, G. R. (1977). *Am. Miner.* **62**, 151–157.

[bb19] Gruehn, R. & Glaum, R. (2000). *Angew. Chem. Int. Ed.* **39**, 692–716. 10760846

[bb20] Hoggins, J. T., Swinnea, J. S. & Steinfink, H. (1983). *J. Solid State Chem.* **47**, 278–283.

[bb21] Hu, G.-R., Xiao, Z.-W., Peng, Z.-D., Du, K. & Deng, X.-R. (2008). *J. Cent. S. Univ. Technol.* **15**, 531–534.

[bb22] Isupov, A. V. (2002). *Ferroelectrics* **274**, 203–283.

[bb23] Khan, F. B., Bharuth-Ram, K. & Friedrich, H. B. (2010). *Hyperfine Interact.* **197**, 317–323.

[bb24] Kobashi, D., Kohara, S., Yamakawa, J. & Kawahara, A. (1997). *Acta Cryst.* C**53**, 1523–1525.

[bb25] Koziskova, J., Hahn, F., Richter, J. & Kožíšek, J. (2016). *Acta Chim. Slovaca* **9**, 136–140.

[bb26] Krishnamachari, N. & Calvo, C. (1972). *Acta Cryst.* B**28**, 2883–2885.

[bb27] Lee, G.-H., Seo, S.-D., Shim, H.-W., Park, K.-S. & Kim, D.-W. (2012). *Ceram. Int.* **38**, 6927–6930.

[bb28] Liang, Y., Zeng, G., Hao, X., Zhao, K., Liu, X., Guo, J., Ren, X., Sun, Q., Qiao, Y., Gao, Q. & Liang, E. (2024). *Appl. Phys. Lett.* **125**, 162201.

[bb29] Liu, S., Gu, C., Wang, H., Liu, R., Wang, H. & He, J. (2015). *J. Alloys Compd.* **646**, 233–237.

[bb30] Lukaszewicz, K. (1967*a*). *Bull. Acad. Pol. Sci. Ser. Sci. Chim.* **15**, 53–57.

[bb31] Lukaszewicz, K. (1967*b*). *Bull. Acad. Pol. Sci. Ser. Sci. Chim.* **15**, 47–51.

[bb32] Mochizuki, Y., Nagamatsu, K., Koiso, H., Isobe, T. & Nakajima, A. (2024). *J. Phys. Chem. Lett.* **15**, 156–164. 10.1021/acs.jpclett.3c02856 PMC10788959 38149933

[bb33] Müller, U. & de la Flor, G. (2024). *Symmetry relationships between crystal structures*, 2nd ed. Oxford University Press.

[bb34] Ong, S. P., Wang, L., Kang, B. & Ceder, G. (2008). *Chem. Mater.* **20**, 1798–1807.

[bb35] Palatinus, L. & Chapuis, G. (2007). *J. Appl. Cryst.* **40**, 786–790.

[bb36] Palatinus, L., Dušek, M., Glaum, R. & El Bali, B. (2006). *Acta Cryst.* B**62**, 556–566. 10.1107/S0108768106010238 16840805

[bb37] Parada, C., Perles, J., Sáez-Puche, R., Ruiz-Valero, C. & Snejko, N. (2003). *Chem. Mater.* **15**, 3347–3351.

[bb38] Petříček, V., Eigner, V., Dušek, M. & Čejchan, A. (2016). *Z. Kristallogr.* **66**, 603–614.

[bb39] Petříček, V., Palatinus, L., Plášil, J. & Dušek, M. (2023). *Z. Kristallogr.* **238**, 271–282.

[bb40] Popović, L., de Waal, D. & Boeyens, J. C. A. (2005). *J. Raman Spectrosc.* **36**, 2–11.

[bb41] Rulmont, A., Cahay, R., Liegeois–Duyckaerts, M. & Tarte, P. (1991). *Eur. J. Solid State Inorg. Chem.* **28**, 207–219.

[bb42] Runciman, W. A., Sengupta, D. & Gourley, J. T. (1973). *Am. Miner.* **58**, 451–456.

[bb43] Salcedo, I. R., Bazaga-García, M., Cuesta, A., Losilla, E. R., Demadis, K. D., Olivera-Pastor, P., Colodrero, R. M. P. & Cabeza, A. (2020). *Dalton Trans.* **49**, 3981–3988. 10.1039/c9dt04210e 31942881

[bb44] Sheldrick, G. M. (2015). *Acta Cryst.* A**71**, 3–8.

[bb45] Stefanidis, T. & Nord, A. G. (1982). *Z. Kristallogr.* **159**, 255–264.

[bb46] Stefanidis, T. & Nord, A. G. (1984). *Acta Cryst.* C**40**, 1995–1999.

[bb47] Stoe (2024). *X-AREA.* Stoe & Cie GmbH, Darmstadt, Germany.

[bb48] Stöger, B., Weil, M. & Dušek, M. (2014). *Acta Cryst.* B**70**, 539–554. 10.1107/S205252061401049X 24892601

[bb49] Tolédano, J.-C., Glazer, A. M., Hahn, Th., Parthé, E., Roth, R. S., Berry, R. S., Metselaar, R. & Abrahams, S. C. (1998). *Acta Cryst.* A**54**, 1028–1033.

[bb50] Wang, B., Duan, Y., Wei, M., Guo, J., Chao, M., Gao, Q., Gao, Y., Li, Z., Xie, J., Liang, E. & Ren, X. (2024). *Results Phys.* **61**, 107785.

[bb51] Weil, M. & Stöger, B. (2010). *Acta Cryst.* B**66**, 603–614. 10.1107/S0108768110040802 21099023

[bb52] Zachariasen, W. H. (1930). *Z. Kristallogr.* **73**, 1–6.

[bb53] Zeng, G., Gao, Y., Guo, J., Qiao, Y., Liang, E. & Gao, Q. (2023). *Ceram. Int.* **49**, 39843–39849.

