# Supplementary material for: New structural insights into Fe2P2O7 – unravelling an unresolved dispute and three reversible phase transitions
Source: IUCrJ. 2025 Oct 9;12(Pt 6):670–82. doi: 10.1107/S2052252525007547 (PMC12573923; doi:10.1107/S2052252525007547)
Supplement: Supplementary file 2 [file m-12-00670-sup2.pdf]

IUCrJ

Volume 12 (2025)

Supporting information for article:

New structural insights into Fe2P2O7 – unravelling an unresolved dispute and three reversible phase transitions

Berthold Stöger, Matthias Weil, Robert Glaum, Karla Fejfarová, Vaclav Petříček, Michal Dušek, Eugen Libowitzky and Ekkehard Füglein

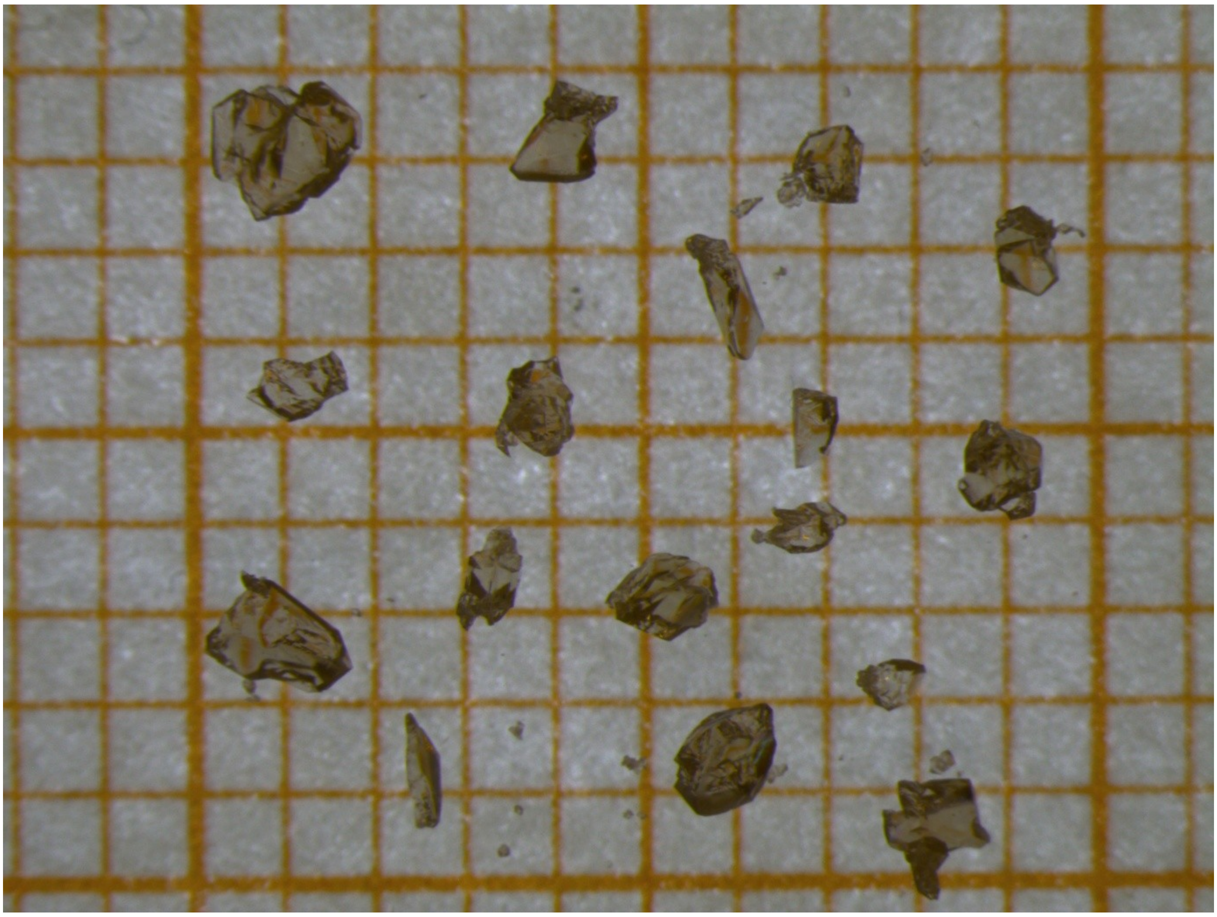

Figure S1 Microphotograph of a selection of obtained $Fe_2P_2O_7$ crystals grown via chemical vapour transport reactions. The distance between the small-scale bars is 1 mm.

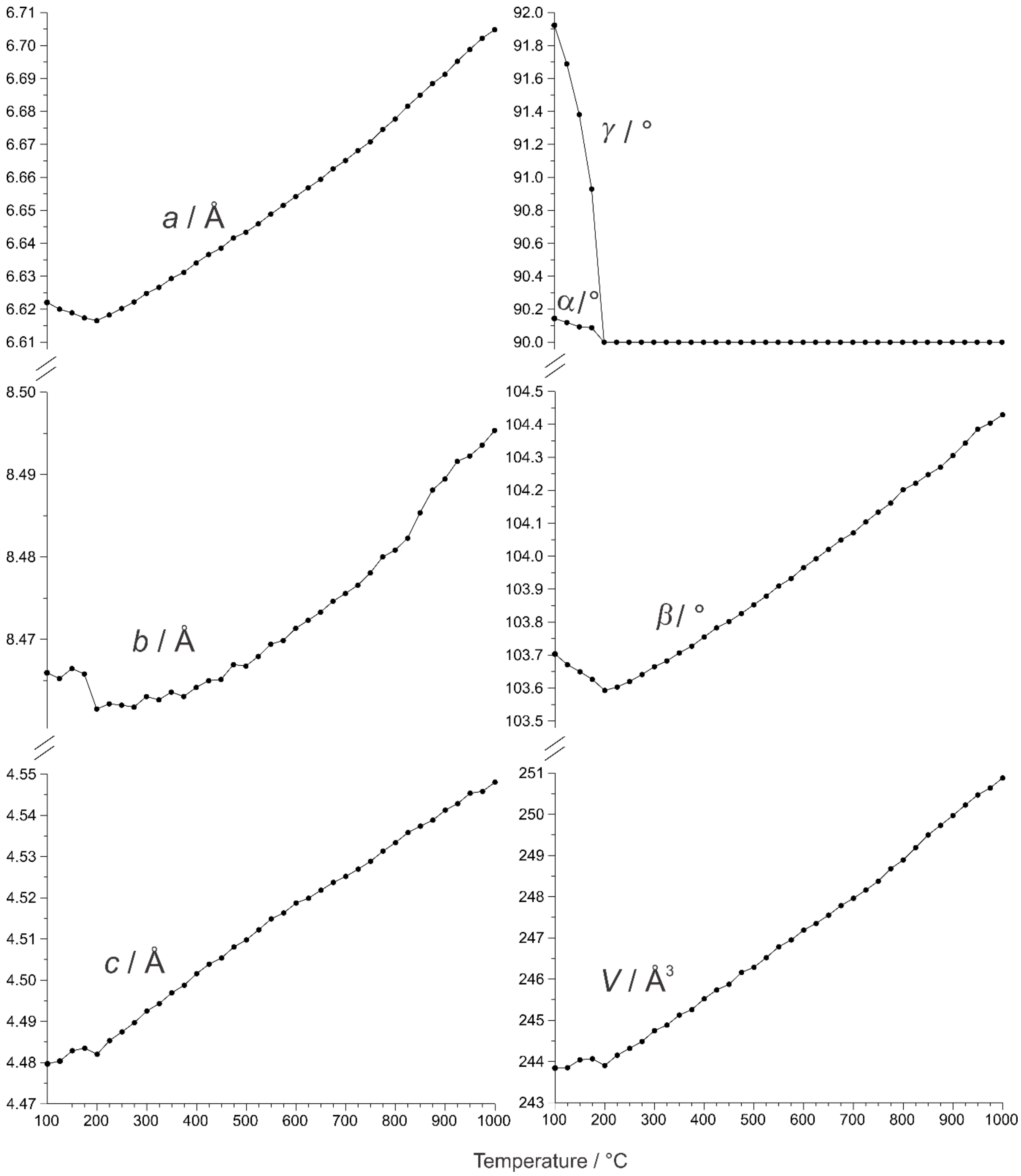

Figure S2 Evolution of lattice parameters for the *α*3- and *β*-modifications with temperature. The phase transition α → *β* is clearly visible by the kink in the individual curves and is particularly pronounced for the *γ* angle.

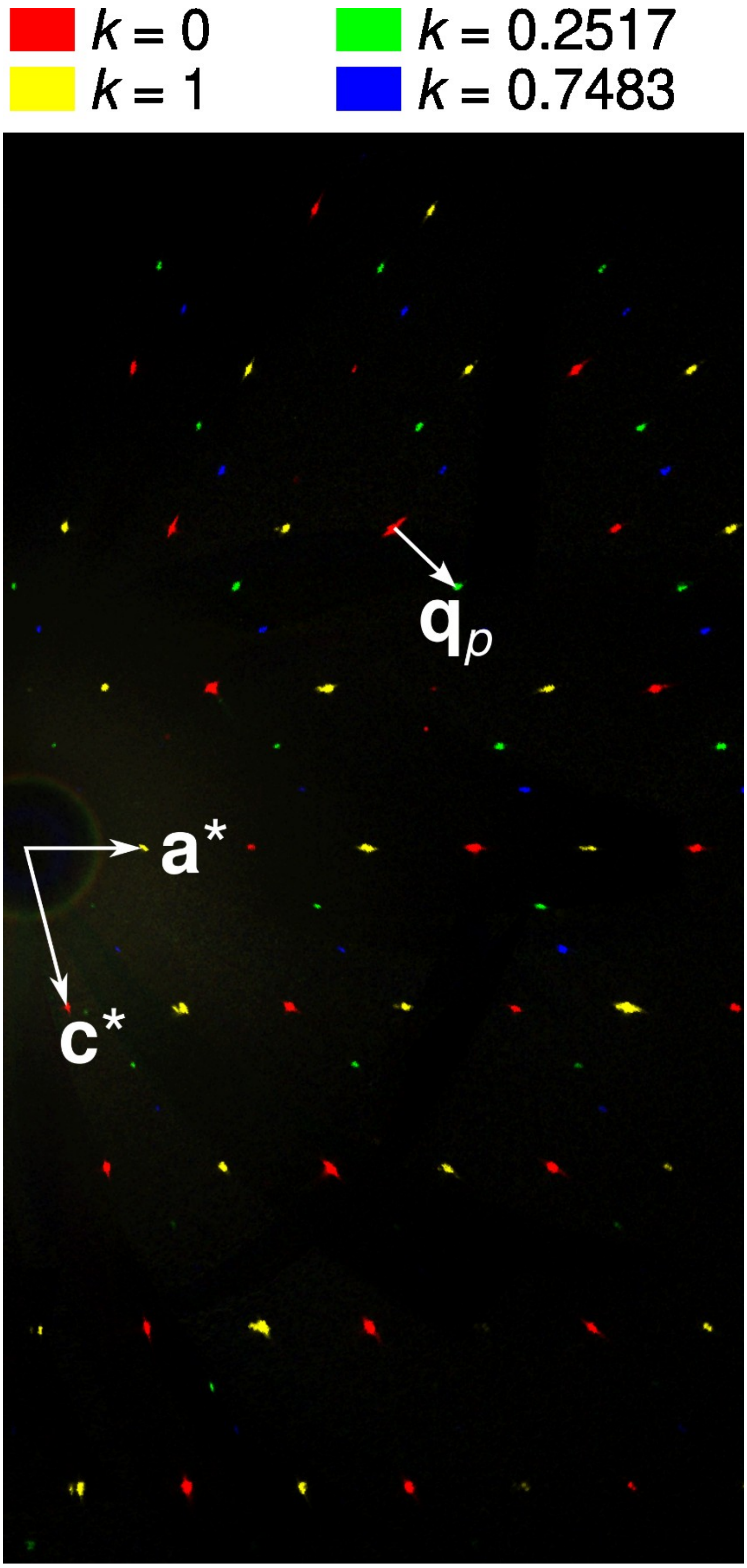

Figure S3 Composite image created by synthesizing planes at $k = 0$ (red), $k = 1$ (yellow), $k = 0$ .2517 (green) and $k = 0.7483$ (blue) of reciprocal space of α_2-$Fe_2P_2O_7$ (27 °C measurement) reconstructed from intensity data, showing planes of main reflections and first order satellites. The reciprocal basis and the vector **q** are indicated by white arrows. A subscript "p" indicates an out-of-plane vector **q** (b* component of 0.2517).

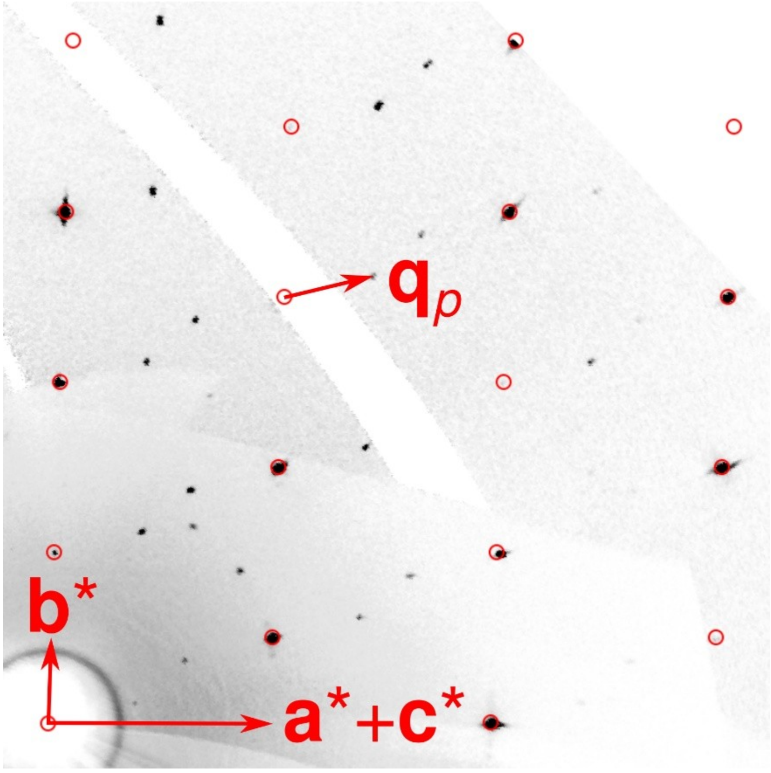

Figure S4 Slab of reciprocal space parallel to a*+c* and b* with 0.02 Å^{-1} thickness in orthogonal projection with averaging of voxel intensities, showing main reflections (expected positions marked by red circles) and first order satellites of α_2-$Fe_2P_2O_7$ (27 °C measurement). Lattice vectors and the modulation wave vector **q** are indicated by red arrows, whereby a subscript "p" indicates a (slightly) out-of-plane modulation wave vector.
